# Multi-omics analysis of lactylation as a prognostic signature: A pan-cancer study

**DOI:** 10.1016/j.gendis.2025.101769

**Published:** 2025-07-12

**Authors:** Xinning Liu, Yanping Wang, Yufeng Cao, Jinbao Zong

**Affiliations:** a Central Laboratory, Clinical Laboratory and Qingdao Key Laboratory of Immunodiagnosis, Qingdao Traditional Chinese Medicine Hospital, Qingdao Hiser Hospital Affiliated of Qingdao University, Qingdao, Shandong, 266034, China; b Department of Pediatrics, Qingdao Traditional Chinese Medicine Hospital, Qingdao Hiser Hospital Affiliated of Qingdao University, Qingdao, Shandong, 266034, China; c Department of Oncology, Qingdao Traditional Chinese Medicine Hospital, Qingdao Hiser Hospital Affiliated of Qingdao University, Qingdao, Shandong, 266034, China

Tumor cells undergo metabolic reprogramming to enhance biomass uptake, which is crucial for their survival and proliferation.^1^^,^^2^ Lactate, traditionally considered as an energy substrate, promotes tumor growth by inducing histone lactylation, which alters gene expression and chromatin structure.^3^ This lactylation process influences tumor progression through immunosuppression, metabolic reprogramming, and macrophage polarization.^4^^,^^5^ However, how lactylation drives cancer development remains unclear. This study explores lactylation in tumor progression across 32 cancer types. A pan-cancer prognostic model based on lactylation-related genes (LRGs) was constructed using TCGA RNA transcriptome data. Cox, Lasso regression, and Kaplan–Meier survival analysis were employed to establish a survival prognostic score (Lscore). High lactylation levels were linked to poor patient prognosis, through influencing immune cell infiltration and promoting tumor metastasis by accelerating the cell cycle. Single-cell RNA sequencing revealed that lactylated cells were predominant in advanced tumor stages, with pathway analysis suggesting connections between lactylation, metastasis, immune cell phagocytosis, and lipid metabolism. This research deepens our understanding of tumor biology through lactylation, offering valuable insights for the development of novel therapeutic strategies and personalized treatment approaches. The workflow for constructing the LRGs signature of pan-cancer is depicted in Figure 1A.

**Figure 1:**
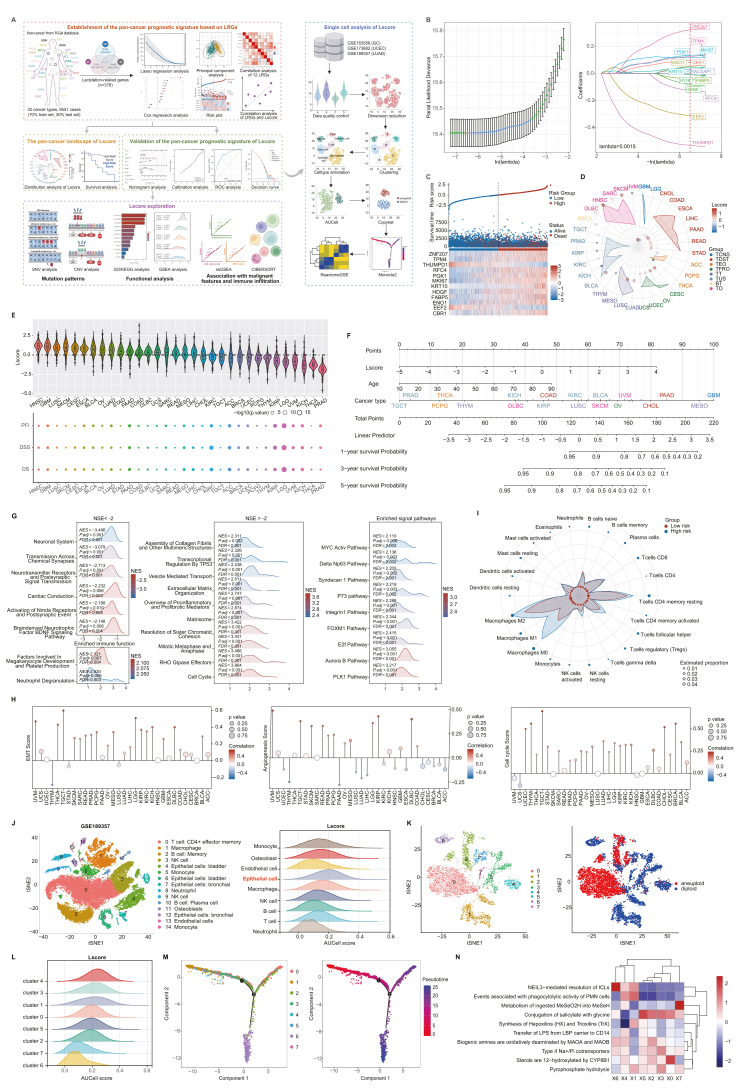
Multi-omics analysis of lactylation as a prognostic signature. **(A)** The workflow for constructing the LRGs signature of pan-cancer. **(B)** Establishment of the pan-cancer prognostic signature based on LRGs and Lasso coefficient spectrum of prognostic gene screening. **(C)** Risk score distribution, survival status of patients, and heatmap of prognostic gene distribution in the TCGA training cohort. **(D)** The distribution of Lscore in different tumor types. **(E)** Univariate Cox regression analysis was performed to assess the association between Lscore and patient prognosis in 32 tumor types using data from TCGA. The analysis included overall survival (OS), disease-specific survival (DSS), and progression-free survival (PFS). *p* < 0.05 indicates a statistically significant association between Lscore and the respective survival outcome. **(F)** Nomogram with certain characteristics for predicting prognosis in the TCGA pan-cancer cohort. **(G)** Gene sets enriched with the lowest NES score (NES < −2), the highest NES score (NES > −2), signaling pathways, and immune cell function. **(H)** The correlation of Lscore with malignant features (epithelial–mesenchymal transition, angiogenesis, and cell cycle) of the individual cancer. **(I)** The 22 immune infiltrations of the high-risk and low-risk lactylation groups were calculated by the CIBERSORT algorithm. **(J)** Single-cell RNA sequencing analysis validated the LRGs-related signature. All cells were visualized with t-SNE, and major cell types were colored according to annotated unsupervised clustering. AUCell score of major cell types was used to evaluate the Lscore of each cell, and the quantification of AUCell was performed. **(K)** t-SNE plot of epithelial cells colored by clusters. The cells within the red were malignant cells based on copy number variations inferred by the CopyKAT algorithm. **(L)** AUCell score of major cell types was used to evaluate the Lscore of each cell, and the quantification of AUCell was performed. **(M)** The developmental trajectory analysis was annotated by sample, and pseudotime was predicted by Monocle2. **(N)** Functional enrichment analysis using the “ReactomeGSA” package. LRGs, lactylation-related genes; TCNS, tumors of the central nervous system; TT, thoracic tumors; TDST, tumors of the digestive system; BT, breast tumor; TUS, tumors of the urinary system and male genital organs; TFRO, tumors of female reproductive organs; TEO, tumors of endocrine organs; TO, tumors of others; NES, normalized enrichment score; t-SNE, t-distributed stochastic neighbor embedding.

To generate a pan-cancer prognostic signature based on LRGs, we identified 20 LRGs using univariate Cox regression (Table S1), and then applied LASSO regression (Fig. 1B), which led to the identification of 14 genes for multivariate Cox regression analysis. Ultimately, 12 LRGs were identified as independent prognostic factors, and a risk signature was established based on the coefficients of these 12 LRGs (Fig. S1A). Notably, genes like fatty acid binding protein 5 (FABP5), enolase 1 (ENO1), phosphoglycerate kinase 1 (PGK1), and marker of proliferation Ki-67 (MKI67), which have been extensively studied in cancer biology, were included. FABP5 encodes the fatty acid-binding protein that plays important roles in fatty acid transport and metabolism. ENO1 and PGK1, linked to aerobic glycolysis, are correlated with the cancer prognosis. Conversely, genes like replication factor C subunit 4 (RFC4), carbonyl reductase 1 (CBR1), keratin 10 (KRT10), zinc finger protein 207 (ZNF207), THUMP domain containing 1 (THUMPD1), and tropomyosin 4 (TPM4) are less explored in oncology. Patients in the TCGA cohort were stratified into high-risk (Lscore > 0, *n* = 5046) and low-risk (Lscore < 0, *n* = 4504) lactylation groups based on the prognostic Lscore (Fig. S1B). To test the robustness of this classification, we recalculated the optimal cutoff using the surv_cutpoint function, which yielded −0.159. Survival analyses in both training and test cohorts using this new cutoff still showed significant differences between groups (*p* < 0.001), confirming the stability of the Lscore-based model (results not shown). Survival analysis revealed better overall survival in the low-risk lactylation groups (Fig. 1C). Expression of prognostic genes also varied between the two groups, with strong positive correlations observed between MKI67 and RFC4, ENO1 and PGK1, heparin-binding growth factor (HDGF) and MKI67, and HDGF and RFC4 in both high-risk and low-risk lactylation groups (Fig. S1C). Interestingly, a high correlation between FABP5 and KRT10 was only observed in the high-risk group. RFC4 and ZNF207 showed stronger positive correlations with other LRGs in the low-risk group compared with the high-risk group.

To validate the prognostic value of the Lscore, survival analysis of the TCGA training cohort showed that high Lscore correlated with poor disease-specific survival, overall survival, and progression-free survival across multiple cancer types (*p* < 0.001), with consistent findings in the test cohort (Fig. S2A). The predominant risk type in cancers was positively correlated with the Lscore value distribution (Fig. S2B). A positive Lscore was observed in cancers, such as head and neck squamous cell carcinoma, lung squamous cell carcinoma, esophageal carcinoma, bladder urothelial carcinoma, ovarian serous cystadenocarcinoma, lung adenocarcinoma, stomach adenocarcinoma, and pancreatic adenocarcinoma (Fig. 1D). Many studies have associated lactate accumulation to molecular changes in these cancers. However, few studies have explored the potential role of lactated modifications in the development of gallbladder cancer, bladder urothelial carcinoma, and stomach adenocarcinoma. In addition to chest and digestive system tumors, female reproductive organ cancers, such as cervical squamous cell carcinoma and endocervical adenocarcinoma, and ovarian serous cystadenocarcinoma, also exhibited high Lscore. Conversely, male genital organ tumors, including prostate adenocarcinoma and testicular germ cell tumors, demonstrated a low Lscore. This may be related to the need for acidification in the female reproductive organs. Besides, tumors from the thymus, prostate, and thyroid also showed higher Lscore, potentially linked to hormone secretion. The univariate Cox model and Kaplan–Meier survival curves revealed better survival outcomes with lower Lscore in various cancers (Fig. 1E; Fig. S2C). The univariate Cox regression analysis revealed the association between Lscore and overall survival of the TCGA training and test cohort patients across the 32 cancer types (Fig. S3A). The results were further confirmed in the two external cohorts (Fig. S3B). The predictive power of Lscore was confirmed with a nomogram incorporating age, cancer types, and Lscore by predicting 1-, 3-, and 5-year overall survival (Fig. 1F). The nomogram model, which integrates the Lscore with age and cancer type, demonstrated better predictive performance than the Lscore alone (the value for the area under the curve: 0.78 *vs*. 0.71) in both the TCGA training and test cohorts (Fig. S4A–S4C). Decision curve analysis further confirmed its utility for nomogram prediction (Fig. S4D).

We further investigate the mutational landscape of the LRGs-related signature. Higher frequencies of gain and loss mutations in LRGs were observed in uterine carcinosarcoma and ovarian serous cystadenocarcinoma (Fig. S5A and S5B). RFC4, HDGF, and TPM4 showed higher gain mutation frequencies, while eukaryotic translation elongation factor 2 (EEF2) and ENO1 had higher loss mutation frequencies across ovarian serous cystadenocarcinoma, uterine carcinosarcoma, and cholangiocarcinoma. We also noted increased mutation frequencies in uterine corpus endometrial carcinoma, skin cutaneous melanoma, and colon adenocarcinoma (Fig. S5C). Analyzing mutation types and frequencies revealed that missense and nonsense mutations were prevalent across most cancer types (Fig. S5D). LRG mutations were equally frequent in both high-risk and low-risk groups (Fig. S5E), suggesting that LRGs with distinct mutation profiles may not be directly involved in lactylation.

To understand the mechanisms behind poor prognosis in high-risk patients, we identified 140 differentially expressed genes between the high- and low-risk groups (Table S3). Gene Ontology (GO) and Kyoto Encyclopedia of Genes and Genomes (KEGG) pathway enrichment analyses revealed that lactylation-related differentially expressed genes were involved in processes such as microtubule binding, cytoskeletal motor activity, nuclear division, chromosome segregation, cell cycle, IL-17 signaling pathway, and progesterone-mediated oocyte maturation (Table S4; Fig. S6A and S6B). Gene Set Enrichment Analysis (GSEA) highlighted hallmark pathways associated with the cell cycle (normalized enrichment score = 3.9) and RHO GTPase effectors (normalized enrichment score = 3.5) in the high-risk group. Additional gene sets enriched in the high-risk group included polo-like kinase 1 (PLK1), aurora B, early 2 factor (E2f), and forkhead box M1 (FOXM1) pathway, as well as immune-related pathways (Table S5; Fig. 1G).

We further investigated the correlation between lactylation and malignancy features such as the cell cycle, epithelial–mesenchymal transition, and angiogenesis (Fig. S7A–S7C). The Lscore showed pronounced positive correlations with the cell cycle (*r* = 0.57, *p* < 0.0001) and epithelial–mesenchymal transition (*r* = 0.27, *p* < 0.0001), and negative correlations with angiogenesis (*r* = −0.12, *p* < 0.0001) (Fig. S8A–S8C). In the high-risk lactylation group, correlations between these features were stronger than in the low-risk group (*p* < 0.001) (Fig. 1H; Fig. S8D–S8F). It is noteworthy that the degree of lactylation was found to be more strongly correlated with the cell cycle. Immune infiltration analysis revealed that the high-risk group had higher levels of M0 (0.034 *vs*. 0.017), M1 (0.02 *vs*. 0.01), and M2 (0.043 *vs*. 0.034) macrophages compared with the low-risk group (Fig. 1I; Fig. S8G).

Finally, we explored lactylation at the single-cell level by integrating three single-cell RNA-sequencing datasets (gastric cancer, lung adenocarcinoma, and uterine corpus endometrial carcinoma). The marker genes' expression in these cell subpopulations was illustrated in Figure S9A. Higher lactylation levels were observed in macrophages, epithelial cells, and endothelial cells, while lower levels were found in B cells, CD4 T cells, and natural killer cells (Fig. 1J; Fig. S9B and S9C). Re-clustering of epithelial cells revealed higher lactylation in the aneuploid group compared with the diploid group (Fig. 1K,L; Fig. S9D and S9E). Monocle 2 trajectory analysis linked high lactylation to advanced stages (Fig. 1M; Fig. S9F). ReactomeGSA pathway analysis suggested that cluster 0 and cluster 2, identified as aneuploid groups, exhibited high expression in hydroxylated sterols and low expression in phagocytic activity of polymorphonuclear leukocytes and Nei-like 3 (NEIL3)-mediated resolution of induced interstrand crosslinks (Fig. 1N; Fig. S9G).

In conclusion, our study highlights the significant role of lactylation in cancer prognosis and suggests that lactylation-based biomarkers can be used for predicting survival outcomes across various cancer types. Further research is needed to explore the therapeutic potential of targeting lactylation in cancer treatment.

## CRediT authorship contribution statement

**Xinning Liu:** Validation, Data curation, Methodology, Conceptualization. **Yanping Wang:** Data curation, Conceptualization. **Yufeng Cao:** Validation, Visualization, Conceptualization. **Jinbao Zong:** Writing – original draft, Writing – review & editing.

## Data availability

Data are available in a public, open-access repository. All raw data, code, and materials are available from the corresponding author upon request. The datasets (GSE163558, GSE173682, and GSE189357) supporting the conclusions of this article are available in the TCGA (https://www.cancer.gov/ccg/research/genome-sequencing/tcga) and GEO (https://www.ncbi.nlm.nih.gov/geo/) database repository.

## Funding

This work has been supported by the Shandong Provincial Natural Science Foundation of China (No. ZR2024QH145), the Qingdao Medical Health Research Project (Shandong, China) (No. 2024-WJKY040), theQingdao Traditional Chinese Medicine Science Foundation (Shandong, China) (No. 2022-zyym07), the Shandong Provincial Traditional Chinese Medicine Science Foundation (Shandong, China) (No. M−2023176), and the Qingdao Outstanding Health Professional Development Fund.

## Conflict of interests

The authors declared no competing interests.
