# Supplementary material for: Multi-omics analysis of lactylation as a prognostic signature: A pan-cancer study
Source: Genes Dis. 2025 Jul 12;13(2):101769. doi: 10.1016/j.gendis.2025.101769 (PMC12664809; doi:10.1016/j.gendis.2025.101769)
Supplement: Multimedia component 1 [file mmc1.docx]

**Summary of SI**

**Supplemental Methods**

**Data collection**

To provide a comprehensive overview of the LRGs signature across pan-cancer, we collected transcriptomic data, including mRNA expression profiles, copy number variations (CNVs), and single-nucleotide variations (SNVs), from The Cancer Genome Atlas (TCGA) database (<https://tcga-data.nci.nih.gov/tcga/>), involving 9551 cases across 32 tumour types. The TCGA dataset was randomly divided into a training cohort (70% of the total samples) and a test cohort (30% of the total samples). Subsequently, 378 LRGs obtained from previously reported studies ^1-3^ were displayed in **supplementary Table S2**. Two external cohorts, CGGA (n= 657) and GSE42127 (n= 133), were used for validation of the predictive performance of the Lscore signature. The CGGA cohort is a publicly available dataset of glioma patients, containing transcriptomic and clinical data from Chinese patients. The GSE42127 cohort is a NSCLC dataset comprising patients who underwent curative resection at MD Anderson Cancer Center between 1996 and 2007. with available transcriptomic and clinical data.

**Construction of prognostic LRGs signature**

The univariate Cox regression was adopted to identify the association of LRG signature with the overall survival (OS) of patients in the TCGA pan-cancer training series, a p value of 2.07E-25 was considered as the screening cut-off criteria. To further obtain more accurate independent prognostic genes and construct a risk model, the application of the Least Absolute Shrinkage Selection Operator (LASSO) analysis, based on the R package “glmnet” and multivariate Cox regression analysis was adopted. The LRG-related risk model and prognostic risk score (Lscore = each prognostic gene’s expression × corresponding coefficient) were constructed using the final set of genes. The Lscore was subsequently standardized across all patients using z-score normalization, and patients were stratified into lactylation high- and low-risk groups based on the standardized Lscore. To verify the robustness of risk stratification, we additionally determined the optimal cutoff value for the Lscore using the surv_cutpoint function from the “survminer” R package. Pearson correlation analyses were applied to explore the correlation pattern of LRGs with each other in the TCGA training cohort. The “survival” R program was used for survival analysis using the Kaplan–Meier (KM) analyses.

**Prognostic potential and accuracy of the LRGs-related signature**

To display risk prediction based on multivariate Cox regression analysis, a prognostic nomogram incorporating the Lscore, and other clinical features was constructed using the “RMS” R package. The calibration plot, a common chart used to evaluate a nomogram’s consistency, was also created using the “RMS” R package. The “timeROC” package was applied to display the receiver operating characteristic (ROC) curves to assess the prognostic capability of the Lscore in predicting survival at 1, 3, and 5 years. The accuracy level of the area under the curve (AUC) can be classified as low (0.5–0.7), moderate (0.7–0.9), and high (above 0.9), with an AUC closer to 1, indicating a superior diagnostic efficacy. The performance of the nomogram was evaluated through decision curve analysis (DCA) by “stdca” R package.

**Function enrichment analysis and gene set enrichment analysis (GSEA)**

The differentially expressed genes (DEGs) between lactylation high- and low-risk groups were analyzed by using the “Limma” R package. Adjusted *p* < 0.05 and |Log2 fold change (FC)| > 2 were used as a threshold for significantly differential expression. Gene Ontology (GO) and Kyoto Encyclopedia of Genes and Genomes (KEGG) annotation analysis were standard methods for enrichment studies. The GO enrichment analysis was annotated to explore the biological significance of the DEGs. The biological process (BP), molecular function (MF), and cellular component (CC) categories of DEGs were performed using a David online tool (https://david.ncifcrf.gov/). KEGG pathway enrichment analysis of DEGs was performed using the KEGG Orthology-Based Annotation System version 3.0 online analysis database (http://39.103.204.200). The two indicators of adj. *p* < 0.05 and false discovery rate (FDR) q < 0.25 were used to evaluate whether the pathways were statistically significant. GSEA analysis was performed to identify enriched gene sets between lactylation high- and low-risk groups. The gene sets of “c2.cp.all.v2022.Hs.symbols.gmt” was used from the Molecular Signatures Database (MSigDB) (<https://www.gsea-msigdb.org/gsea/msigdb/>) ^4^. The “clusterProfiler” R package was employed to conduct the GSEA analysis. The two indicators of adj. *p* < 0.05 and FDR q < 0.25 were used to evaluate whether the gene sets were statistically significant.

**The correlation of Lscore with malignant features and immune cell infiltration**

The gene sets related to angiogenesis ^5^, epithelial-mesenchymal transition (EMT) ^6^, and the cell cycle ^7^ are collected, and the malignant feature scores of the TCGA pan-cancer samples were calculated based on the existing gene sets by the single-sample gene set enrichment analysis (ssGSEA). The correlation between Lscore and other feature scores can be analyzed using spearman's statistical method. |R| ≥ 0.8: highly correlated; 0.5 ≤ |R| < 0.8: moderately correlated; 0.3 ≤ |R| < 0.5: low correlation; |R| < 0.3: weak correlation. Statistical analysis and visualization are performed in R version 4.2.1. The statistical analyses were conducted using the “stats” package and the “car” package, and the analysis results are visualized by correlation scatter plots as well as box plots using gglot2. The immune infiltration in lactylation high- and low-risk groups was calculated by examining the signature of 22 kinds of immune cell expression spectrum matrix using the CIBERSORTx website (https:// cibersortx.stanford.edu/) ^8^.

**scRNA-seq analysis**

The raw scRNA data of 3 GC patients (GSE163558), 5 UCEC patients (GSE173682), and 3 LUAD (GSE189357) patients were downloaded from the GEO database and processed using the “Seurat” R package as described before ^9^. Low-quality cells (100 < nCount < 50000; 100 < features < 5000; > 10% mitochondrial genes) were filtered, and the data were initially normalized. No sample batch correction was performed. Dimensions were reduced using t-distributed stochastic neighbor embedding (t-SNE), and differentially expressed genes for each cluster were found using the Seurat FindMarkers function. 14 (46811 cells), 9 (14143 cells), and 11 (35208 cells) cell clusters, in the GSE189357, GSE163558, and GSE173682 datasets were obtained, respectively, for downstream analysis. Reference-based cell type annotation was generated by “SingleR” function, including CD4 T cell, B cells, NK cell, DC, macrophage, epithelial cell, endothelial cell, and epithelial cells. The marker genes expression in these cell subpopulations were illustrated in **Figure S5**. To identify the benign and malignant epithelial cells, we used the “CopyKAT” R package to estimate the CNVs. This method has been widely validated for identifying malignant cells in solid tumors and does not require predefined reference cell population ^10^. We used the “AUCell” R package to evaluate the Lscore of each cell in the scRNA-seq dataset and score each cell based on the 12 LRGs. The single cell pseudotime and trajectory analyses of epithelial cells were constructed using the “Monocle2” R package. Further detection with the Monocle2 plot pseudotime heatmap function revealed the differentiation progress of cells with default parameters of Monocle ^11^. In addition, we used the “ReactomeGSA” R package for pathway enrichment analysis.

**Statistical analysis**

The statistical analysis was conducted using R software (version 4.2.1) and the corresponding R package. Default parameters were used for parameters that were not shown. The corresponding sections describe the statistical methods used. A significance level of *p* < 0.05 was considered significant, while ns denotes no significance. Additionally, **p* < 0.05, ***p* < 0.01, and ****p* < 0.001.

**Supplemental Figure Legends:**

**Figure S1.** Establishment of the pan-cancer prognostic signature based on LRGs. **(A)**. The model genes and their gene coefficient. (Lscore = CBR1 exp * 0.1 + EEF2 exp * -0.324 + ENO1 exp * 0.056 + FABP5 exp * -0.051 + HDGF exp * -0.106 + KRT10 exp * 0.049 + MKI67 exp * 0.161 + PGK1 exp * 0.125 + RFC4 exp * -0.134 + THUMPD1 exp * -0.547 + TPM4 exp * 0.131 + ZNF207 exp * 0.353) **(B)**. Distributions of lactylation low- and high-risk patients of the TCGA training cohort were plotted in the PCA. **(C)**. The correlations between 12 prognostic gene expressions in the TCGA training and test cohort were calculated using the Pearson method.

**Figure S2.** The pan-cancer landscape of Lscore across 32 cancer types. **(A)** The KM survival curves illustrate the OS, DSS, and PFI in lactylation high- and low-risk group of TCGA training (n= 6686) and test (n= 2865) cohort. **(B)** The proportion of lactylation high- and low-risk samples distributed across tumours. **(C)**. The KM survival curves illustrate the OS in lactylation high- and low-risk groups of ACC (n= 79), KIRC (n= 531), KIRP (n= 287), PAAD (n= 177), LIHC (n= 365), LGG (n= 511), UCEC (n= 530), MESO (n= 85), LUAD (n= 502), and UVM (n= 80).

**Figure S3.** **(A)** The univariate Cox regression analysis of the association between Lscore and OS of the TCGA training and test cohort patients across the 32 cancer types was shown in the forest plots. **(B)** The KM and ROC curves depicting the predictive performance of the Lscore signature for 1-, 3-, and 5-year overall survival in two external cohorts CGGA (n= 657) and GSE42127 (n= 133).

**Figure S4.** Validation of the pan-cancer prognostic signature based on LRGs. **(A)** Calibration curves of the nomogram model (incorporating Lscore, age, and cancer type) for predicting 1-, 3-, and 5-year overall survival in the TCGA cohort. **(B-C)** ROC curves depicting the predictive performance of the nomogram model and Lscore-only model for 1-, 3-, and 5-year overall survival in TCGA training and test cohort. **(D)**. Decision curve analysis for the nomogram and Lscore-only model in the TCGA training and test cohorts.

**Figure S5.** Mutation patterns in the LRGs-related signature in TCGA pan-cancer. CNV frequencies of the 12 LRGs in the 32 tumour types. Red **(A)** and blue **(B)** colors indicate a CNV gain and loss, respectively. **(C)** The SNV frequency of the LRGs in pan-cancers from TCGA. **(D)** Oncoplot displaying the somatic landscape of 12 LRGs in pan-cancer. **(E)** Oncoplot displaying the somatic landscape of 12 LRGs in BLCA, UCEC, COAD, and STAD.

**Figure S6.** GO/KEGG analysis of DEGs between lactylation high- and low-risk groups. **(A)** Chord plot depicting the relationship between identified genes and GO terms. BP: biological process, CC: cellular component, MF: molecular function. **(B)** Chord plot depicting the relationship between genes and KEGG pathways.

**Figure S7.** The correlation between lactylation and angiogenesis **(A)**, cell cycle **(B)**, and EMT **(C)** in TCGA pan-cancer.

**Figure S8.** Association of the LRGs-related signature with malignant features and immune infiltration in TCGA pan-cancer. **(A-C)** The correlation of Lscore with malignant features of the TCGA pan-cancer. **(D-F)** The correlation of Lscore with malignant features of the individual cancer. **(G)** The estimated proportion of M0, M1, and M2 macrophages in the lactylation high- and low-risk group of individual cancer.

**Figure S9.** scRNA-seq analysis validated the LRGs-related signature. **(A)** The marker genes expression in cell subpopulations were illustrated in bubble diagram. **(B-C)** t-SNE plot of all cells colored by major cell types according to annotated unsupervised clustering of GSE163558 and GSE173682. AUCell score of major cell types was used to evaluate the Lscore of each cell, and the quantification of AUCell was performed. **(D)** t-SNE plot of epithelial cells colored by clusters of GSE163558. The cells within the red were malignant cells based on CNVs inferred by the CopyKAT algorithm. **(E)** AUCell score of major cell types was used to evaluate the Lscore of each cell in GSE163558, and the quantification of AUCell was performed. **(F)** The developmental trajectory analysis annotated by sample and pseudotime predicted by Monocle2 of GSE163558. **(G)** Functional enrichment analysis using the “ReactomeGSA” package of GSE163558.

**Supplemental Table legends**

**Table S1.** The univariate Cox regression analysis perform in the TCGA pan-cancer training cohort, and top 20 genes with p value < 2.07E-25 were displayed in Table S1.

**Table S2.** LRGs obtained from previously reported studies were displayed in Table S2.

**Table S3.** The DEGs between the high- and low-risk groups determined with cutoff criterion as adj. *p* < 0.05 and |Log2 fold change (FC)| > 2 were identified in Table S3.

**Table S4.** The GO and KEGG pathway enrichment combined with Log2FC value analysis of DEGs between the high- and low-risk groups were shown in Table S4.

**Table S5.** The GSEA between the high- and low-risk groups with cutoff criterion as adj. *p* < 0.05 and FDR q < 0.25 were performed, the enriched gene sets were showed in supplementary Table S5.

**Reference**

1. Jiao Y, Ji F, Hou L, Lv Y, Zhang J. Lactylation-related gene signature for prognostic prediction and immune infiltration analysis in breast cancer. *Heliyon*. Feb 15 2024;10(3):e24777. doi:10.1016/j.heliyon.2024.e24777

2. Wu J, Lv Y, Hao P, et al. Immunological profile of lactylation-related genes in Crohn's disease: a comprehensive analysis based on bulk and single-cell RNA sequencing data. *J Transl Med*. Mar 23 2024;22(1):300. doi:10.1186/s12967-024-05092-z

3. Cheng Z, Huang H, Li M, Liang X, Tan Y, Chen Y. Lactylation-Related Gene Signature Effectively Predicts Prognosis and Treatment Responsiveness in Hepatocellular Carcinoma. *Pharmaceuticals (Basel)*. Apr 25 2023;16(5)doi:10.3390/ph16050644

4. Liberzon A, Birger C, Thorvaldsdottir H, Ghandi M, Mesirov JP, Tamayo P. The Molecular Signatures Database (MSigDB) hallmark gene set collection. *Cell Syst*. Dec 23 2015;1(6):417-425. doi:10.1016/j.cels.2015.12.004

5. Masiero M, Simoes FC, Han HD, et al. A core human primary tumor angiogenesis signature identifies the endothelial orphan receptor ELTD1 as a key regulator of angiogenesis. *Cancer Cell*. Aug 12 2013;24(2):229-41. doi:10.1016/j.ccr.2013.06.004

6. Yu TJ, Ma D, Liu YY, et al. Bulk and single-cell transcriptome profiling reveal the metabolic heterogeneity in human breast cancers. *Mol Ther*. Jul 7 2021;29(7):2350-2365. doi:10.1016/j.ymthe.2021.03.003

7. Sanchez-Vega F, Mina M, Armenia J, et al. Oncogenic Signaling Pathways in The Cancer Genome Atlas. *Cell*. Apr 5 2018;173(2):321-337 e10. doi:10.1016/j.cell.2018.03.035

8. Newman AM, Liu CL, Green MR, et al. Robust enumeration of cell subsets from tissue expression profiles. *Nat Methods*. May 2015;12(5):453-7. doi:10.1038/nmeth.3337

9. Butler A, Hoffman P, Smibert P, Papalexi E, Satija R. Integrating single-cell transcriptomic data across different conditions, technologies, and species. *Nat Biotechnol*. Jun 2018;36(5):411-420. doi:10.1038/nbt.4096

10. Gao R, Bai S, Henderson YC, et al. Delineating copy number and clonal substructure in human tumors from single-cell transcriptomes. *Nat Biotechnol*. May 2021;39(5):599-608. doi:10.1038/s41587-020-00795-2

11. Qiu X, Mao Q, Tang Y, et al. Reversed graph embedding resolves complex single-cell trajectories. *Nat Methods*. Oct 2017;14(10):979-982. doi:10.1038/nmeth.4402
