# Supplementary figures and images for: Multi-omics analysis of lactylation as a prognostic signature: A pan-cancer study
Source: Genes Dis. 2025 Jul 12;13(2):101769. doi: 10.1016/j.gendis.2025.101769 (PMC12664809; doi:10.1016/j.gendis.2025.101769)

Figure S1

A

| Model genes | Gene coefficient |
|-------------|------------------|
| ZNF207      | 0.353            |
| MKI67       | 0.161            |
| TPM4        | 0.131            |
| PGK1        | 0.125            |
| CBR1        | 0.1              |
| ENO1        | 0.056            |
| KRT10       | 0.049            |
| FABP5       | -0.051           |
| HDGF        | -0.106           |
| RFC4        | -0.134           |
| EEF2        | -0.324           |
| THUMPD1     | -0.547           |

B

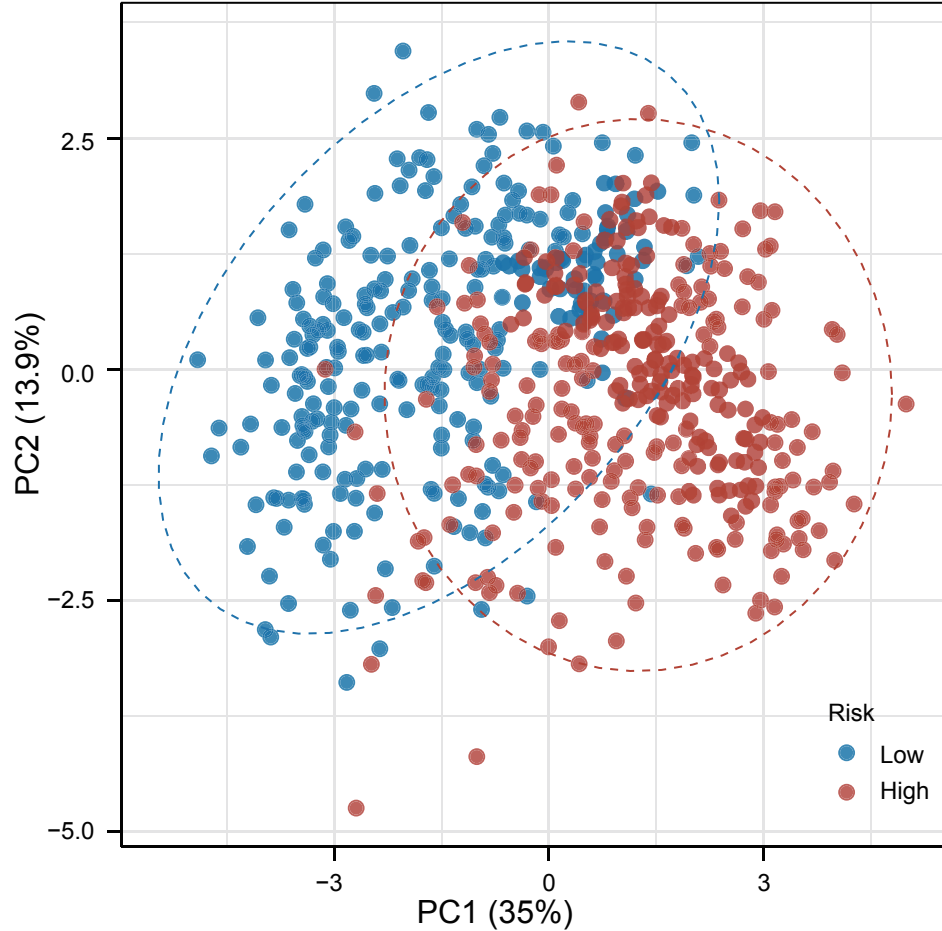

C

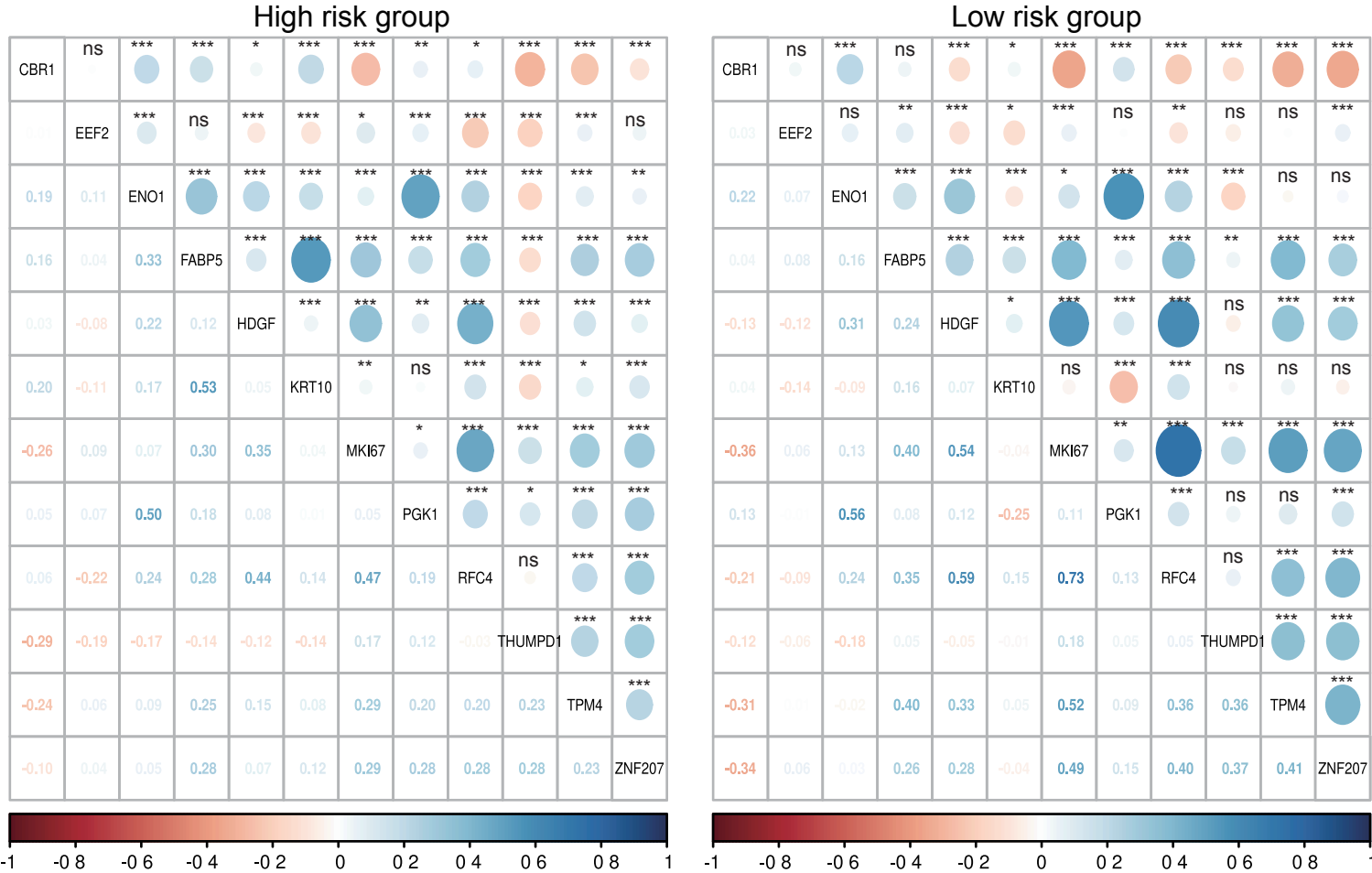

Supplement: Multimedia component 2 [file mmc2.pdf]

**Figure S2**

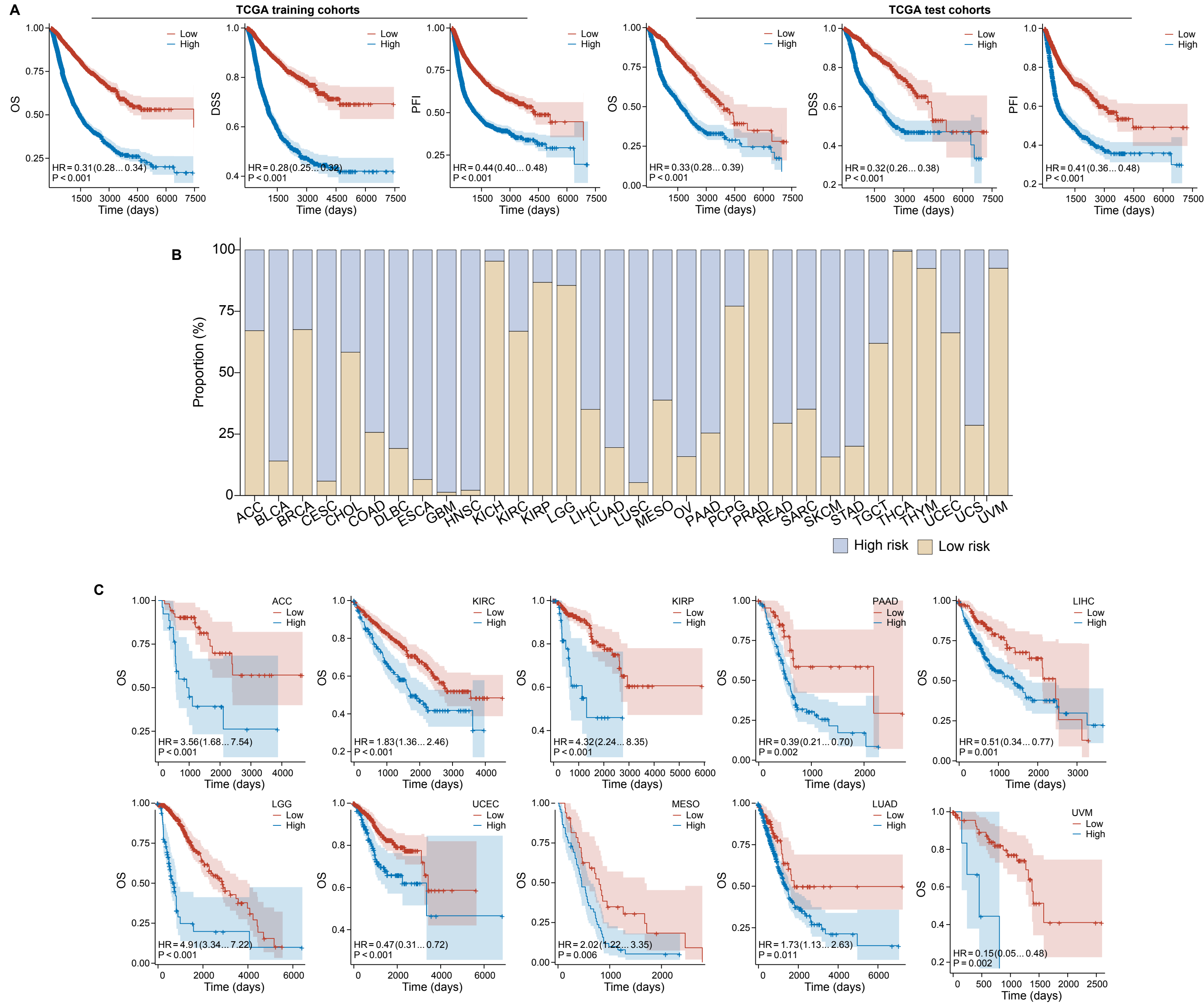

Supplement: Multimedia component 3 [file mmc3.pdf]

Figure S3

A

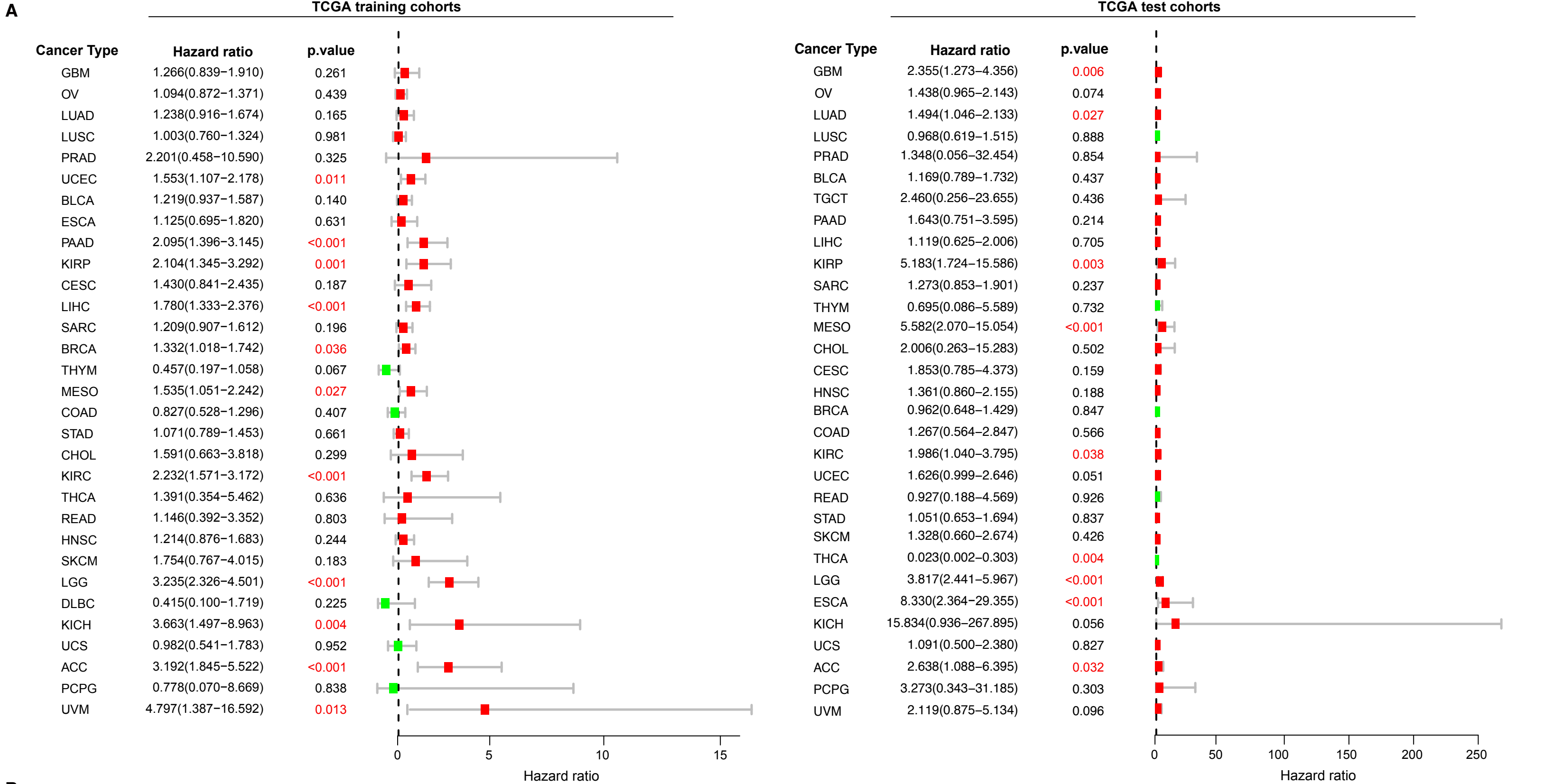

B

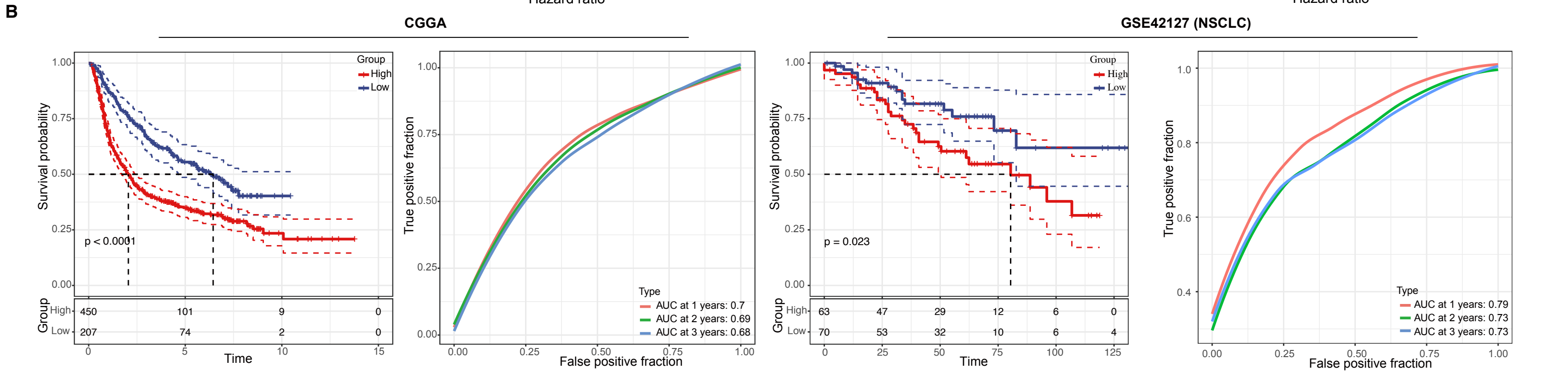

Supplement: Multimedia component 4 [file mmc4.pdf]

Figure S4

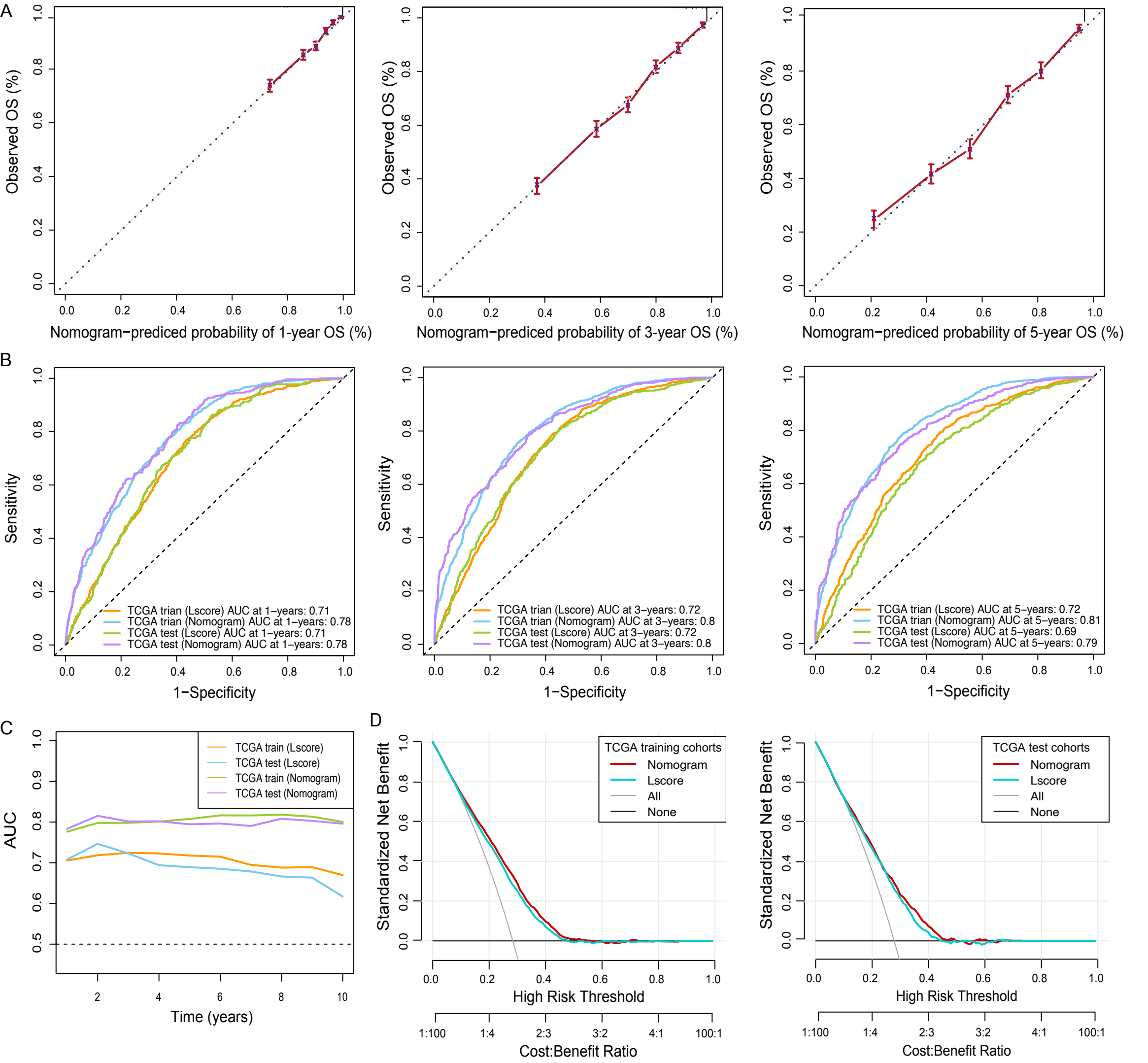

Supplement: Multimedia component 5 [file mmc5.pdf]

Figure S5

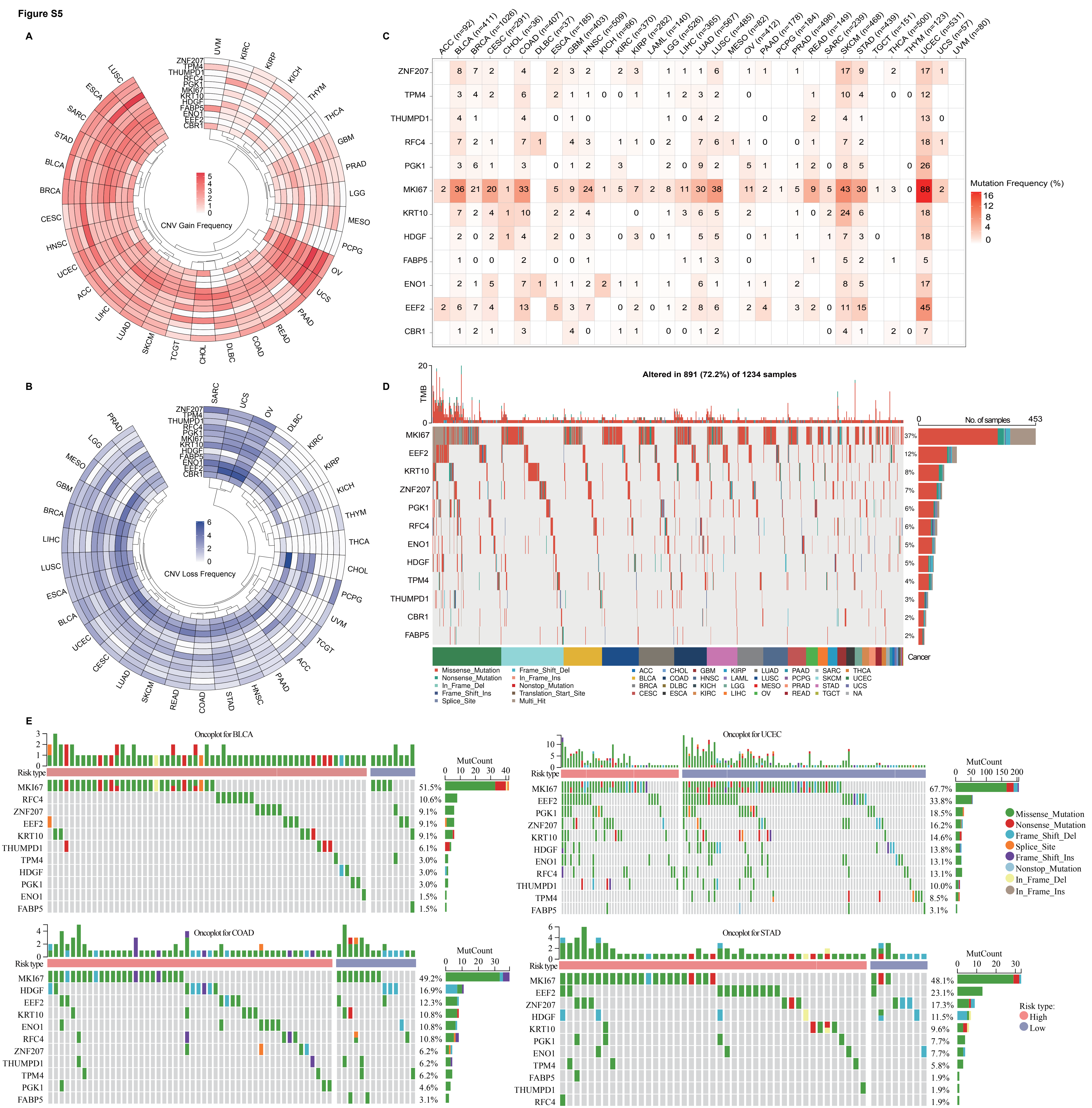

Supplement: Multimedia component 6 [file mmc6.pdf]

Figure S6

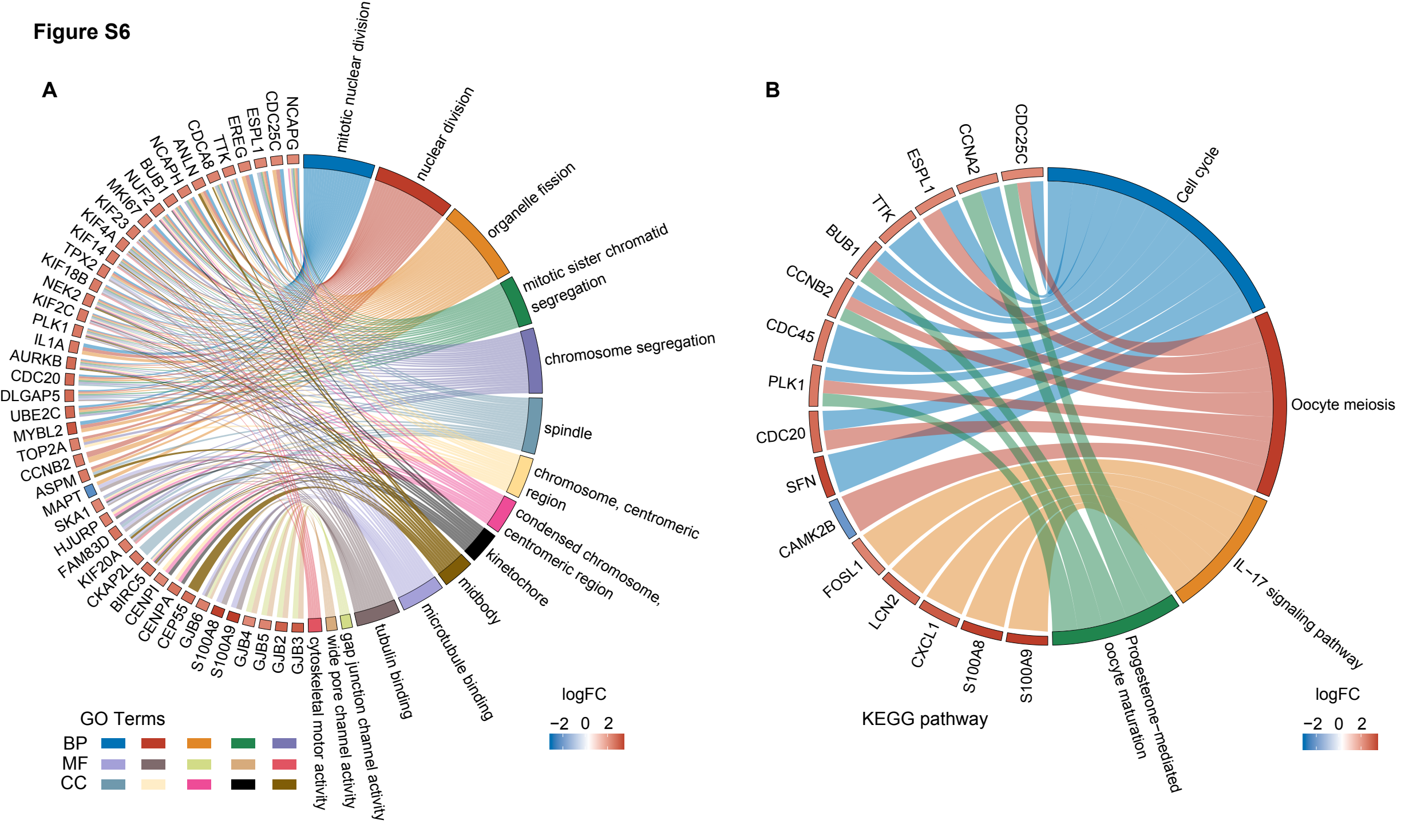

Supplement: Multimedia component 7 [file mmc7.pdf]

**Figure S7****A**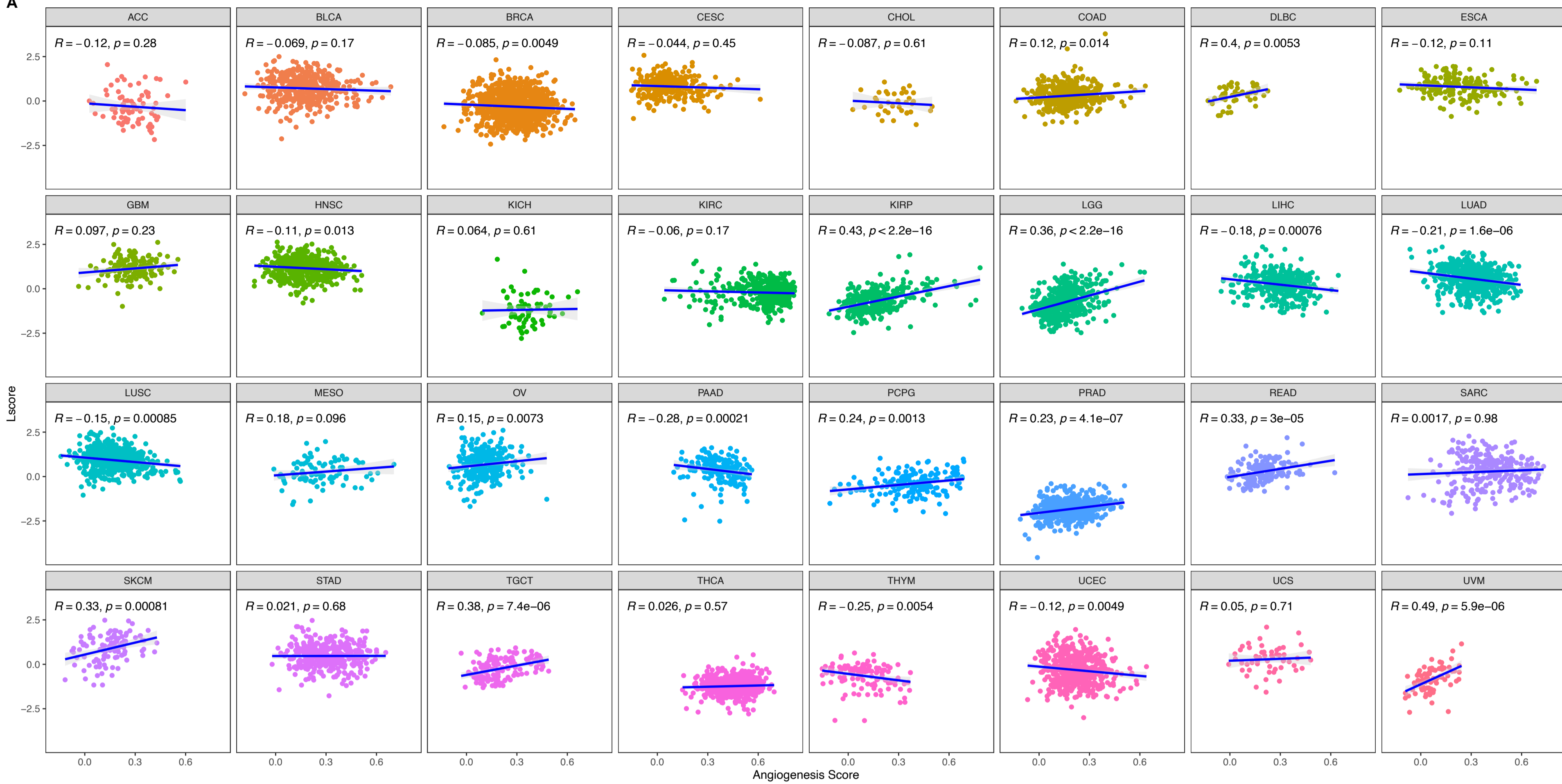**B**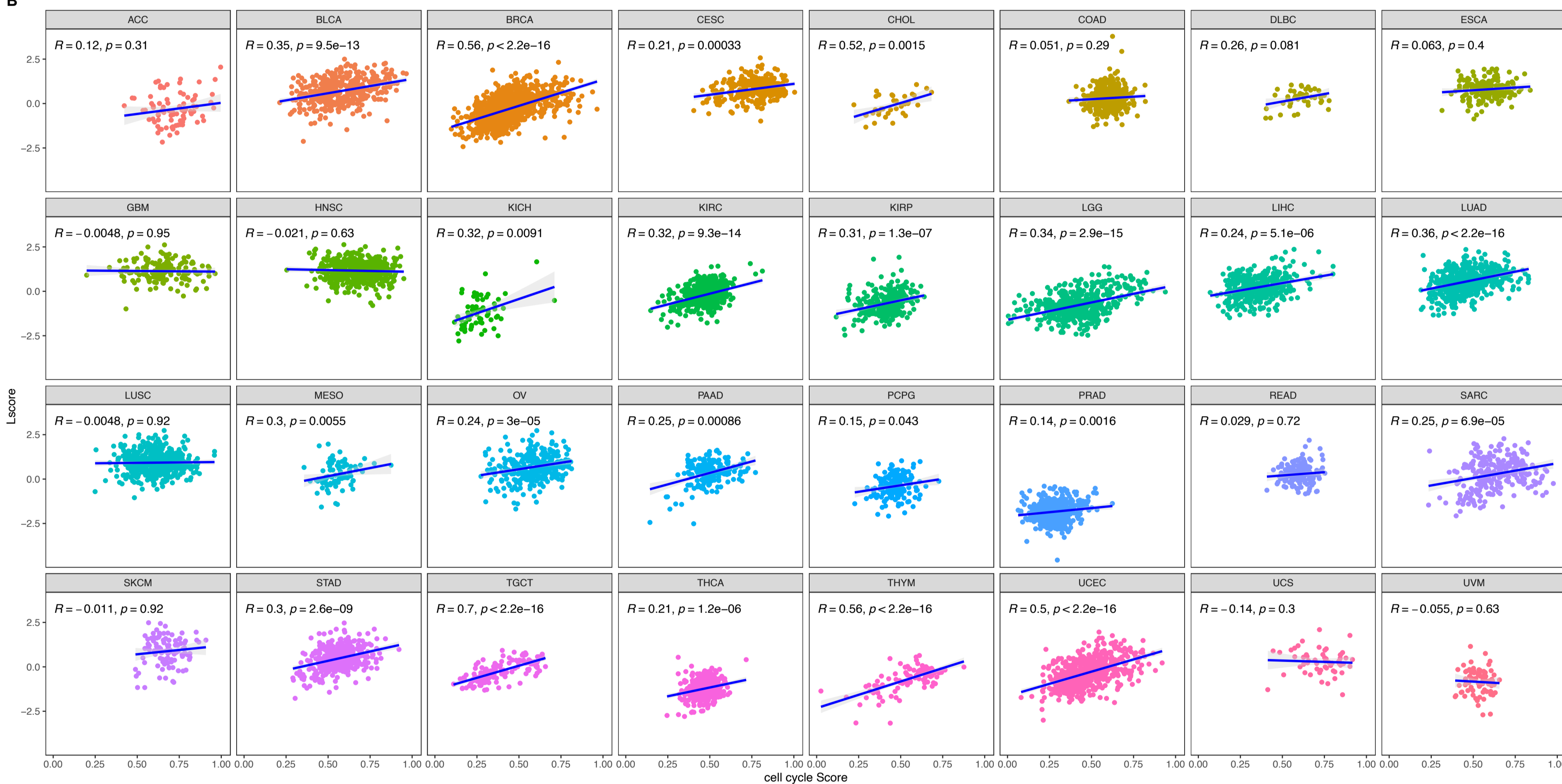**C**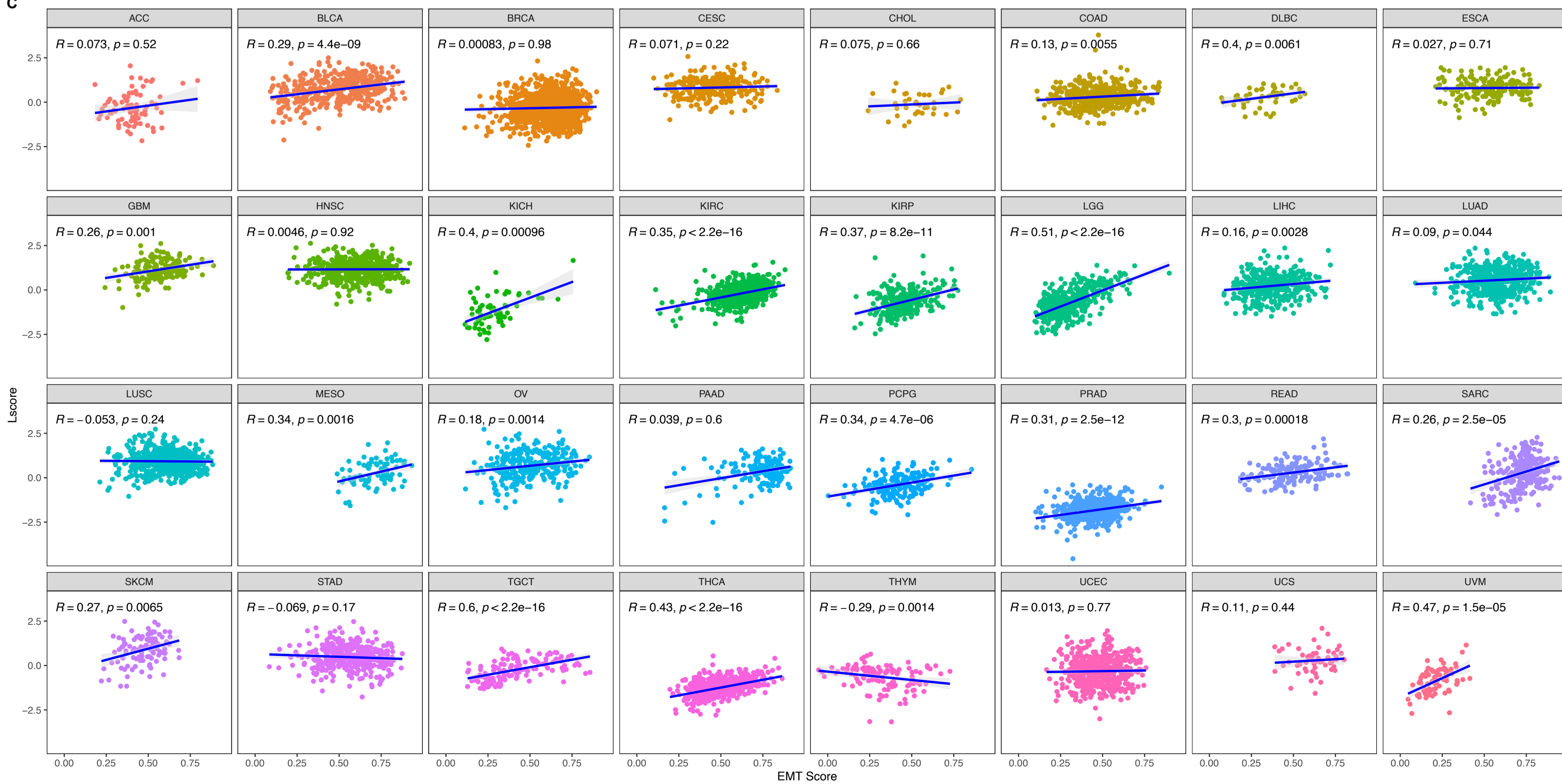

Supplement: Multimedia component 8 [file mmc8.pdf]

Figure S8

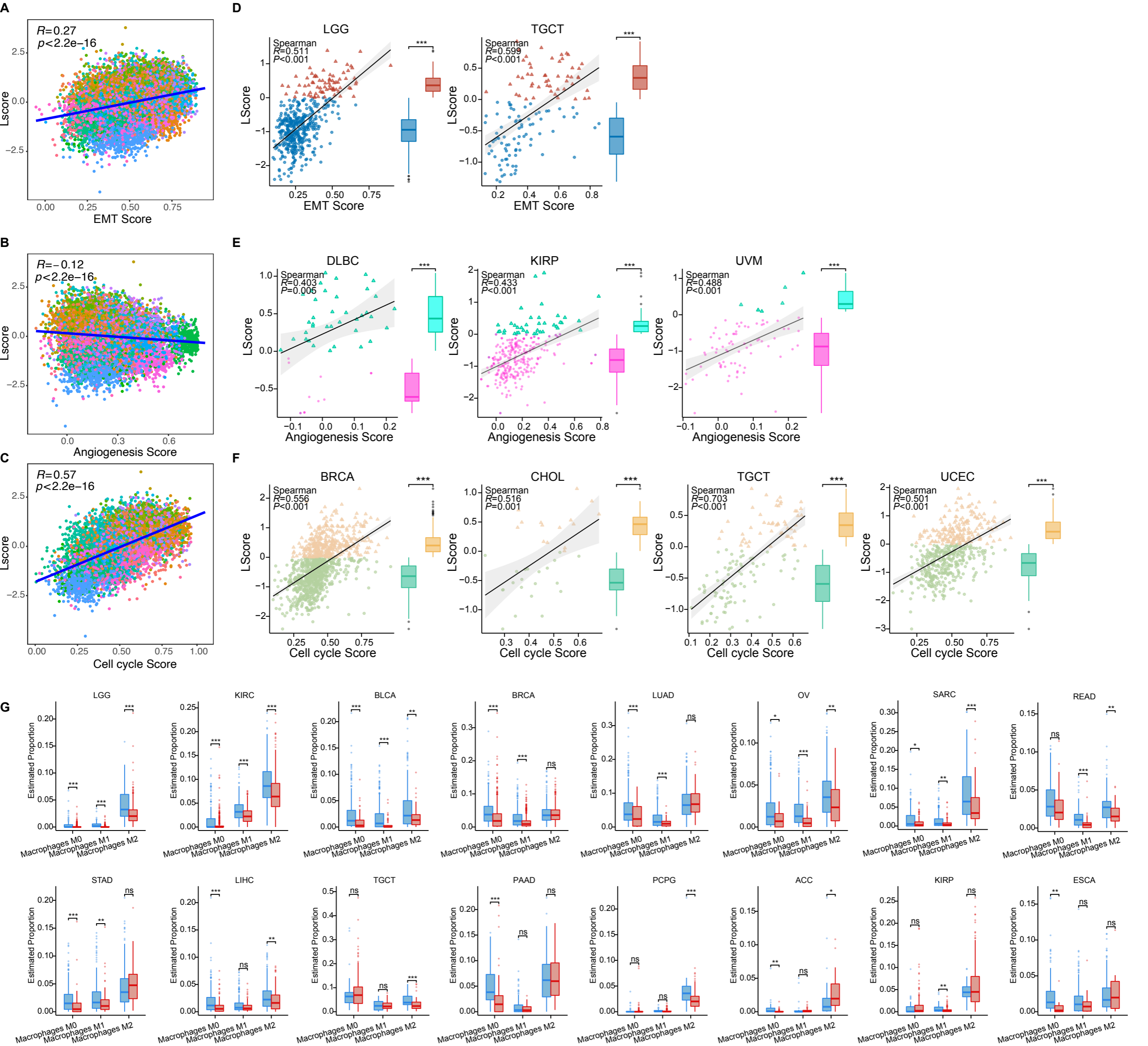

Supplement: Multimedia component 9 [file mmc9.pdf]

Figure S9

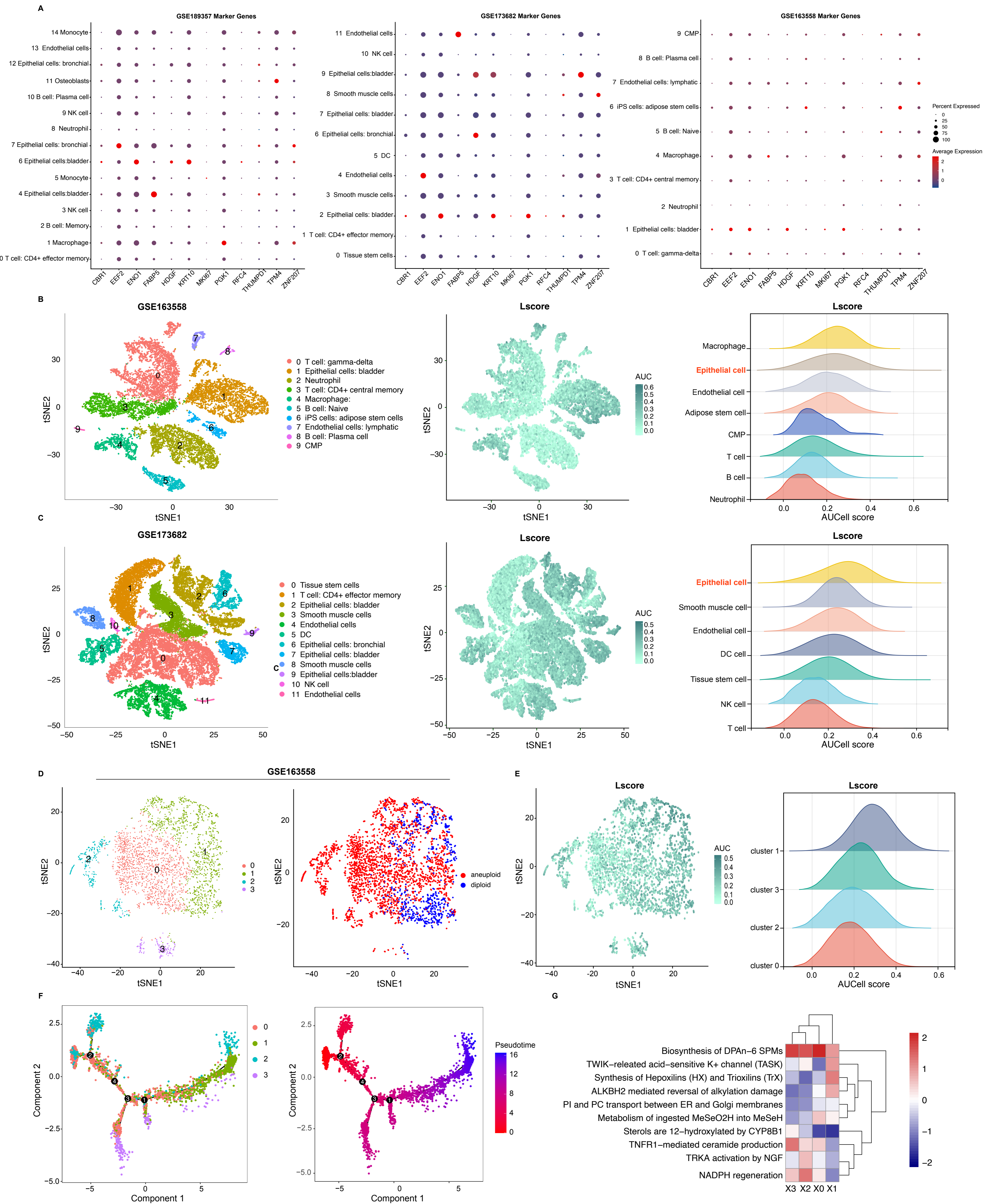

Supplement: Multimedia component 10 [file mmc10.pdf]
