# Supplementary material for: Multi-omics analysis of lactylation as a prognostic signature: A pan-cancer study
Source: Genes Dis. 2025 Jul 12;13(2):101769. doi: 10.1016/j.gendis.2025.101769 (PMC12664809; doi:10.1016/j.gendis.2025.101769)
Supplement: Multimedia component 11 [file mmc11.pdf]

Table S1: The univariate Cox regression analysis perform in the TCGA pan-cancer training cohort, and top 20 genes with p value < 2.07E-25 were selected.

Gene	P value	HR	Low.95.CI	High.95.CI
**THUMPD1**	2.75E-65	0.564	0.528	0.602
**KIF2C**	7.05E-57	1.220	1.191	1.251
**CCNA2**	6.40E-56	1.242	1.209	1.276
**RACGAP1**	8.11E-52	1.331	1.283	1.382
**MKI67**	5.19E-50	1.211	1.181	1.242
**RAN**	8.32E-48	1.561	1.470	1.657
**CCT5**	9.48E-48	1.533	1.447	1.624
**PSMA7**	4.16E-43	1.515	1.428	1.607
**ENO1**	7.65E-41	1.361	1.301	1.424
**PGK1**	1.10E-40	1.387	1.322	1.455
**RFC4**	2.94E-40	1.294	1.246	1.344
**EEF2**	1.89E-38	0.656	0.616	0.700
**FABP5**	2.65E-34	1.112	1.093	1.131
**TPM4**	1.43E-33	1.302	1.247	1.359
**KRT10**	5.13E-32	1.164	1.135	1.194
**HDGF**	3.45E-31	1.437	1.352	1.528
**ZNF207**	8.96E-31	1.954	1.744	2.190
**IFI16**	1.83E-26	1.188	1.151	1.226
**CBR1**	1.74E-25	1.191	1.153	1.231
**GAPDH**	2.07E-25	1.302	1.239	1.368
