# Supplementary material for: Multi-omics analysis of lactylation as a prognostic signature: A pan-cancer study
Source: Genes Dis. 2025 Jul 12;13(2):101769. doi: 10.1016/j.gendis.2025.101769 (PMC12664809; doi:10.1016/j.gendis.2025.101769)
Supplement: Multimedia component 12 [file mmc12.pdf]

Supplemental Table S2

PMID 38318076 (22)	PMID 38521905 (30)	PMID 37242427 (327)	Merge (378)
ARID3A	SLC16A7	ACAT2	ACAT2
CCNA2	SLC16A3	ACIN1	ACIN1
DDX39A	SLC16A1	ADAR	ADAR
EHMT2	SLC16A8	ADNP	ADNP
FABP5	SLC5A8	AHNAK	AHNAK
G6PD	SLC5A12	ALB	ALB
H2AX	EMB	ALDH1A1	ALDH1A1
HMGA1	PER2	ALDOA	ALDOA
KIF2C	SLC25A12	ALDOB	ALDOB
MKI67	TP53	ALYREF	ALYREF
PFKP	TIGAR	ARGLU1	ARGLU1
PKM2	PFKFB2	ARID1A	ARID1A
RACGAP1	LDHC	ARID3A	ARID3A
RFC4	LDHB	ARID3B	ARID3B
STMN1	LDHA	ARPP19	ARPP19
TKT	HIF1A	BCLAF1	BCLAF1
EFNA3	PNKD	BOLA2	BOLA2
VCAN	LDHD	BRD4	BRD4
PLOD2	PARK7	BTF3	BTF3
HBB	HAGH	BZW1	BZW1
NUP50	GLO1	BZW2	BZW2
STC1	SIRT3	C19orf53	C19orf53
	SIRT2	CACYBP	CACYBP
	SIRT1	CALD1	CALD1
	HDAC8	CALM1	CALM1
	HDAC3	CALML5	CALML5
	HDAC2	CALR	CALR
	HDAC1	CBR1	CBR1
	CREBBP	CBX3	CBX3
	EP300	CBX5	CBX5
		CCNA2	CCNA2
		CCT5	CCT5
		CD2BP2	CD2BP2
		CDC5L	CDC5L
		CDV3	CDV3
		CDV3	CDV3
		CDYL	CDYL
		CEBPZ	CEBPZ
		CFDP1	CFDP1
		CHD4	CHD4
		CHERP	CHERP
		CNN2	CNN2
		CNN3	CNN3
		COPS4	COPS4
		CRABP2	CRABP2
		CSRP1	CSRP1
		CSRP2	CSRP2
		CWC15	CWC15
		DDX17	DDX17
		DDX18	DDX18
		DDX21	DDX21
		DDX39A	DDX39A
		DDX39B	DDX39B
		DDX3X	DDX3X

		DDX41	DDX41
		DDX42	DDX42
		DDX46	DDX46
		DDX5	DDX5
		DECR1	DECR1
		DFFA	DFFA
		DHRS7	DHRS7
		DHX16	DHX16
		DHX9	DHX9
		EAF1	EAF1
		ECHDC1	ECHDC1
		EDF1	EDF1
		EEF1A1	EEF1A1
		EEF1G	EEF1G
		EEF2	EEF2
		EHMT2	EHMT2
		EIF3D	EIF3D
		EIF3J	EIF3J
		EIF4G1	EIF4G1
		EIF4G2	EIF4G2
		EIF4H	EIF4H
		EMG1	EMG1
		ENO1	ENO1
		ENSA	ENSA
		FABP5	FABP5
		FAM50A	FAM50A
		FKBP3	FKBP3
		FLYWCH2	FLYWCH2
		FUBP1	FUBP1
		G6PD	G6PD
		GAPDH	GAPDH
		GATAD2A	GATAD2A
		GATAD2B	GATAD2B
		GFAP	GFAP
		GIGYF2	GIGYF2
		GTF2F1	GTF2F1
		GTF2I	GTF2I
		H1-2	H1-2
		H1-3	H1-3
		H1-5	H1-5
		H2AFV	H2AFV
		H2AFZ	H2AFZ
		H2AJ	H2AJ
		H2AX	H2AX
		H2AZ1	H2AZ1
		H2AZ2	H2AZ2
		H2BC13	H2BC13
		H2BC14	H2BC14
		H2BC18	H2BC18
		H2BC5	H2BC5
		H2BU1	H2BU1
		H3-3A	H3-3A
		H3C1	H3C1
		H3C15	H3C15
		H4C1	H4C1
		HCFC1	HCFC1

		HDAC1	HDAC1
		HDAC2	HDAC2
		HDGF	HDGF
		HDGFL2	HDGFL2
		HEXIM1	HEXIM1
		HIST1H1C	HIST1H1C
		HIST1H2BB	HIST1H2BB
		HIST1H2BD	HIST1H2BD
		HIST1H2BH	HIST1H2BH
		HIST1H2BK	HIST1H2BK
		HIST1H2BL	HIST1H2BL
		HIST1H2BN	HIST1H2BN
		HIST1H2BO	HIST1H2BO
		HIST1H3A	HIST1H3A
		HIST1H4A	HIST1H4A
		HIST2H2BE	HIST2H2BE
		HIST2H2BF	HIST2H2BF
		HISTIH2BH	HISTIH2BH
		HLTF	HLTF
		HMGA1	HMGA1
		HMGB1	HMGB1
		HMGN1	HMGN1
		HMGN2	HMGN2
		HMGN3	HMGN3
		HMGN4	HMGN4
		HNRNPA1	HNRNPA1
		HNRNPC	HNRNPC
		HNRNPD	HNRNPD
		HNRNPF	HNRNPF
		HNRNPH1	HNRNPH1
		HNRNPK	HNRNPK
		HNRNPL	HNRNPL
		HNRNPM	HNRNPM
		HNRNPU	HNRNPU
		HSDL2	HSDL2
		HSPE1	HSPE1
		IARS2	IARS2
		IFI16	IFI16
		IK	IK
		IKZF1	IKZF1
		ILF2	ILF2
		ILF3	ILF3
		IRF2BP2	IRF2BP2
		JMJD1C	JMJD1C
		JPT1	JPT1
		JPT2	JPT2
		KHDRBS1	KHDRBS1
		KHSRP	KHSRP
		KIF2C	KIF2C
		KRT1	KRT1
		KRT10	KRT10
		LAP3	LAP3
		LCP1	LCP1
		LDHB	LDHB
		LEMD3	LEMD3
		LGALS1	LGALS1

		LRPPRC	LRPPRC
		LSP1	LSP1
		MAGOH	MAGOH
		MAGOHB	MAGOHB
		MAP2K4	MAP2K4
		MAPRE1	MAPRE1
		MBD2	MBD2
		MBP	MBP
		MDC1	MDC1
		MKI67	MKI67
		MNDA	MNDA
		MPHOSPH6	MPHOSPH6
		MSN	MSN
		MTA1	MTA1
		MTA2	MTA2
		MYH13	MYH13
		NCDN	NCDN
		NCL	NCL
		NEFL	NEFL
		NHLRC2	NHLRC2
		NOC3L	NOC3L
		NOLC1	NOLC1
		NONO	NONO
		NOP2	NOP2
		NPM1	NPM1
		NSUN2	NSUN2
		NUCKS1	NUCKS1
		NUDT21	NUDT21
		NUDT5	NUDT5
		NUP133	NUP133
		NUP50	NUP50
		PABPC1	PABPC1
		PABPN1	PABPN1
		PAK2	PAK2
		PARP1	PARP1
		PCBP1	PCBP1
		PCBP2	PCBP2
		PCMT1	PCMT1
		PCNP	PCNP
		PDAP1	PDAP1
		PDLIM1	PDLIM1
		PES1	PES1
		PFKM	PFKM
		PFKP	PFKP
		PFN1	PFN1
		PGK1	PGK1
		PHC3	PHC3
		PHF6	PHF6
		PKM2	PKM2
		POLDIP3	POLDIP3
		PPIA	PPIA
		PPIL4	PPIL4
		PPM1G	PPM1G
		PPP1CB	PPP1CB
		PPP1CC	PPP1CC
		PPP1R2B	PPP1R2B

		PRAM1	PRAM1
		PRCC	PRCC
		PRDX1	PRDX1
		PRKDC	PRKDC
		PRPF6	PRPF6
		PSMA7	PSMA7
		PSMC1	PSMC1
		PSME3IP1	PSME3IP1
		PTBP1	PTBP1
		PTMA	PTMA
		RACGAP1	RACGAP1
		RALYL	RALYL
		RAN	RAN
		RANBP2	RANBP2
		RB1	RB1
		RBM10	RBM10
		RBM14	RBM14
		RBM17	RBM17
		RBM25	RBM25
		RBM39	RBM39
		RBMX	RBMX
		RCC2	RCC2
		RECQL	RECQL
		RFC1	RFC1
		RFC4	RFC4
		RIMS1	RIMS1
		RPA1	RPA1
		RPL13	RPL13
		RPL14	RPL14
		RPL22	RPL22
		RPL24	RPL24
		RPL29	RPL29
		RPL5	RPL5
		RPS11	RPS11
		RPS23	RPS23
		RPS27A	RPS27A
		RRP1B	RRP1B
		RSL1D1	RSL1D1
		S100A11	S100A11
		S100A4	S100A4
		S100A6	S100A6
		SAFB	SAFB
		SARNP	SARNP
		SATB1	SATB1
		SET	SET
		SF3A1	SF3A1
		SF3B1	SF3B1
		SFPQ	SFPQ
		SFPQ	SFPQ
		SH3GL1	SH3GL1
		SMAP	SMAP
		SMARCA5	SMARCA5
		SMARCC1	SMARCC1
		SMARCC2	SMARCC2
		SMC3	SMC3
		SNRPA1	SNRPA1

		SOD1	SOD1
		SPR	SPR
		SPR14	SPR14
		SRP14	SRP14
		SRRM1	SRRM1
		SRRM2	SRRM2
		SSB	SSB
		STMN1	STMN1
		SUB1	SUB1
		SUMO2	SUMO2
		TCOF1	TCOF1
		TERF2	TERF2
		THOC2	THOC2
		THRAP3	THRAP3
		THUMPD1	THUMPD1
		TKT	TKT
		TMA7	TMA7
		TMPO	TMPO
		TMSB4X	TMSB4X
		TOP2B	TOP2B
		TP53	TP53
		TPM4	TPM4
		TPR	TPR
		TRIM28	TRIM28
		TRIR	TRIR
		TSSC4	TSSC4
		U2AF2	U2AF2
		U2SURP	U2SURP
		UBE2E1	UBE2E1
		UBE2M	UBE2M
		UPF1	UPF1
		VARS	VARS
		VIM	VIM
		WAS	WAS
		WBP11	WBP11
		WDR33	WDR33
		WIZ	WIZ
		XPO5	XPO5
		XRCC4	XRCC4
		YLPM1	YLPM1
		ZC3H18	ZC3H18
		ZC3H4	ZC3H4
		ZMYM3	ZMYM3
		ZNF207	ZNF207
		ZNF280C	ZNF280C
		ZNF706	ZNF706
		ZNFX1	ZNFX1
		ZRANB2	ZRANB2
		ZYX	ZYX
			SLC16A7
			SLC16A3
			SLC16A1
			SLC16A8
			SLC5A8
			SLC5A12
			EMB

			PER2
			SLC25A12
			TP53
			TIGAR
			PFKFB2
			LDHC
			LDHB
			LDHA
			HIF1A
			PNKD
			LDHD
			PARK7
			HAGH
			GLO1
			SIRT3
			SIRT2
			SIRT1
			HDAC8
			HDAC3
			HDAC2
			HDAC1
			CREBBP
			EP300
			CCNA2
			DDX39A
			EHMT2
			FABP5
			G6PD
			H2AX
			HMGA1
			KIF2C
			MKI67
			PFKP
			PKM2
			RACGAP1
			RFC4
			STMN1
			TKT
			EFNA3
			VCAN
			PLOD2
			HBB
			NUP50
			STC1
