# Supplementary material for: Multi-omics analysis of lactylation as a prognostic signature: A pan-cancer study
Source: Genes Dis. 2025 Jul 12;13(2):101769. doi: 10.1016/j.gendis.2025.101769 (PMC12664809; doi:10.1016/j.gendis.2025.101769)
Supplement: Multimedia component 13 [file mmc13.pdf]

## Supplemental Table S3

logfc>2, adj<0.05

| | logFC | AveExpr | t | P.Value | adj.P.Val | B |
|---|---|---|---|---|---|---|
| HPN | -2.591396396 | 7.269051822 | -34.40241828 | 1.29E-244 | 1.61E-243 | 549.0846998 |
| MAPT | -2.582650096 | 7.123135599 | -43.1708464 | 0 | 0 | 842.417367 |
| LONRF2 | -2.529550388 | 6.984557239 | -42.56453161 | 0 | 0 | 820.6253051 |
| CAMK2B | -2.371583852 | 5.469611563 | -42.09883502 | 0 | 0 | 804.0299939 |
| AFF3 | -2.237141458 | 6.709357253 | -43.24018096 | 0 | 0 | 844.9226335 |
| LRP2 | -2.223044708 | 5.462480542 | -27.62506074 | 1.12E-161 | 6.98E-161 | 358.348023 |
| KCNJ16 | -2.193111599 | 4.624494195 | -27.31502165 | 3.04E-158 | 1.83E-157 | 350.4564961 |
| FAM189A | -2.153816793 | 6.399984004 | -45.06625468 | 0 | 0 | 911.8621304 |
| PDE8B | -2.140985253 | 5.82204141 | -46.77396715 | 0 | 0 | 976.079461 |
| GALNTL | -2.112846925 | 6.162103383 | -44.72304343 | 0 | 0 | 899.1419173 |
| ZBTB16 | -2.085678624 | 4.908856165 | -41.98004308 | 0 | 0 | 799.8168365 |
| B3GAT1 | -2.07379448 | 5.201711768 | -34.08599888 | 2.02E-240 | 2.45E-239 | 539.4361972 |
| ANKS1B | -2.069703239 | 4.474113484 | -46.20060223 | 0 | 0 | 954.3489645 |
| ZMAT1 | -2.069375702 | 7.440412438 | -57.05813664 | 0 | 0 | 1391.738316 |
| GRIK3 | -2.047284702 | 4.931827954 | -32.63820481 | 1.28E-221 | 1.34E-220 | 496.1872927 |
| GLYATL | -2.004289242 | 4.125337306 | -31.23948004 | 4.51E-204 | 4.02E-203 | 455.8348251 |
| HMGA2 | 2.001511944 | 5.263216034 | 30.29917713 | 1.25E-192 | 1.01E-191 | 429.5177025 |
| NMU | 2.017276959 | 4.440167331 | 37.85203047 | 2.94E-292 | 5.35E-291 | 658.6769914 |
| CXCL17 | 2.017955787 | 5.553812102 | 25.5680227 | 1.59E-139 | 8.12E-139 | 307.4256696 |
| GJB4 | 2.018172015 | 3.924791395 | 38.26576045 | 3.36E-298 | 6.47E-297 | 672.3463979 |
| ORC1L | 2.022287005 | 6.622246316 | 65.58090434 | 0 | 0 | 1766.29274 |
| SGOL1 | 2.035227141 | 5.667762939 | 60.63411236 | 0 | 0 | 1546.107185 |
| NCAPG | 2.038384609 | 8.078099178 | 63.34800678 | 0 | 0 | 1666.028313 |
| CDC25C | 2.042507534 | 5.9383501 | 59.73483985 | 0 | 0 | 1506.878695 |
| CENPI | 2.045631797 | 5.105406516 | 65.3310338 | 0 | 0 | 1755.005674 |
| LYPD3 | 2.05221476 | 7.339649692 | 32.93521955 | 2.01E-225 | 2.17E-224 | 504.9383744 |
| PRR11 | 2.057836709 | 5.704405713 | 55.88236727 | 0 | 0 | 1341.976688 |
| AIM2 | 2.058031619 | 4.766097425 | 45.27765025 | 0 | 0 | 919.7283573 |
| CCNA2 | 2.059155839 | 8.361217988 | 67.54752774 | 0 | 0 | 1855.683198 |
| CLSPN | 2.062977943 | 6.110693638 | 59.19838603 | 0 | 0 | 1483.604418 |
| CDKN3 | 2.06569721 | 6.947209893 | 68.54303068 | 0 | 0 | 1901.29017 |
| FERMT1 | 2.071235248 | 7.775697816 | 33.75902653 | 4.05E-236 | 4.76E-235 | 529.5393249 |
| ESPL1 | 2.072165736 | 7.684309785 | 59.41728652 | 0 | 0 | 1493.089853 |
| CDCA7 | 2.094023609 | 7.806742471 | 45.78150007 | 0 | 0 | 938.5726956 |
| KIAA119 | 2.09766689 | 7.627118107 | 41.34434429 | 0 | 0 | 777.4106461 |
| SKA1 | 2.097743403 | 6.576079983 | 65.22275257 | 0 | 0 | 1750.11954 |
| B3GNT3 | 2.099990873 | 6.869570405 | 30.80290472 | 1.01E-198 | 8.59E-198 | 443.5340348 |
| E2F7 | 2.105543192 | 6.24480177 | 58.69525008 | 0 | 0 | 1461.864159 |
| GJB5 | 2.107515964 | 4.664497223 | 36.42340998 | 4.13E-272 | 6.45E-271 | 612.3282765 |
| EREG | 2.113284515 | 4.112337054 | 36.01489196 | 1.86E-266 | 2.76E-265 | 599.3218761 |
| TTK | 2.114620254 | 7.289311687 | 58.61675563 | 0 | 0 | 1458.480287 |
| CDCA8 | 2.119892123 | 8.133577881 | 68.84485527 | 0 | 0 | 1915.162793 |

| | | | | | | |
|---|---|---|---|---|---|---|
| FOSL1 | 2.122050968 | 6.837509574 | 50.63297028 | 0 | 0 | 1126.562009 |
| ANLN | 2.142964751 | 8.9987228 | 60.35562906 | 0 | 0 | 1533.930932 |
| EXO1 | 2.156769092 | 6.782677916 | 61.76866372 | 0 | 0 | 1595.967682 |
| MCM10 | 2.157926308 | 6.756484559 | 63.30015363 | 0 | 0 | 1663.894754 |
| A2ML1 | 2.163197627 | 4.251537049 | 29.59556053 | 2.97E-184 | 2.22E-183 | 410.2592622 |
| NCAPH | 2.163307758 | 7.559186666 | 66.56197428 | 0 | 0 | 1810.765253 |
| BUB1 | 2.165375691 | 8.114351417 | 64.55830314 | 0 | 0 | 1720.205233 |
| HJURP | 2.165635967 | 7.59480804 | 63.47235019 | 0 | 0 | 1671.575305 |
| NUF2 | 2.166420085 | 7.221803732 | 61.34453327 | 0 | 0 | 1577.281163 |
| IQGAP3 | 2.169478135 | 8.399472029 | 62.34330796 | 0 | 0 | 1621.373343 |
| TNNT1 | 2.169892203 | 5.35588133 | 31.92378505 | 1.40E-212 | 1.36E-211 | 475.3984692 |
| MKI67 | 2.172084823 | 10.15575811 | 62.50493768 | 0 | 0 | 1628.537083 |
| TMEM40 | 2.17915965 | 3.72387734 | 37.43086783 | 2.94E-286 | 5.16E-285 | 644.8749754 |
| PTPRH | 2.182878009 | 5.813175937 | 38.64472659 | 1.10E-303 | 2.21E-302 | 684.9628298 |
| POLQ | 2.188020311 | 6.476949455 | 63.98234464 | 0 | 0 | 1694.372265 |
| PADI3 | 2.189406084 | 3.38347261 | 37.19619195 | 6.15E-283 | 1.06E-281 | 637.2342103 |
| FOXM1 | 2.19766223 | 9.335320245 | 68.26191015 | 0 | 0 | 1888.387835 |
| TOP2A | 2.1976984 | 10.3627282 | 60.59498607 | 0 | 0 | 1544.394941 |
| GJB6 | 2.198931554 | 4.574265218 | 30.29039559 | 1.60E-192 | 1.29E-191 | 429.2750415 |
| CYP4F3 | 2.202905506 | 4.412188644 | 37.36918187 | 2.20E-285 | 3.84E-284 | 642.8630855 |
| ATP10B | 2.205182876 | 5.284043401 | 32.92273507 | 2.91E-225 | 3.13E-224 | 504.5692663 |
| GREM1 | 2.206859137 | 7.543219228 | 34.43393414 | 4.91E-245 | 6.15E-244 | 550.0495144 |
| IGF2BP2 | 2.216953264 | 7.818288021 | 41.4214877 | 0 | 0 | 780.1170405 |
| CCNB2 | 2.219837181 | 8.242669436 | 65.70508483 | 0 | 0 | 1771.908253 |
| MYEOV | 2.220338466 | 4.933395983 | 38.03839203 | 6.27E-295 | 1.18E-293 | 664.8207242 |
| KIF20A | 2.226355698 | 8.100111053 | 63.10020159 | 0 | 0 | 1654.986925 |
| KIF23 | 2.228405893 | 7.991136615 | 66.62535804 | 0 | 0 | 1813.646865 |
| KIF4A | 2.236043306 | 8.035835203 | 68.06281809 | 0 | 0 | 1879.261319 |
| KIF14 | 2.24506676 | 6.735293673 | 63.03812256 | 0 | 0 | 1652.223672 |
| TNS4 | 2.248251855 | 7.05593413 | 28.5964791 | 1.21E-172 | 8.26E-172 | 383.5629478 |
| TPX2 | 2.252226234 | 9.74476238 | 67.91684927 | 0 | 0 | 1872.575877 |
| E2F8 | 2.264434033 | 6.2195567 | 63.35521279 | 0 | 0 | 1666.349654 |
| CKAP2L | 2.271082691 | 6.603999486 | 68.85935231 | 0 | 0 | 1915.829631 |
| KIF18B | 2.276845234 | 7.421062584 | 66.52238828 | 0 | 0 | 1808.966066 |
| TROAP | 2.283142743 | 7.492963417 | 61.76209435 | 0 | 0 | 1595.677823 |
| NEK2 | 2.285239788 | 7.458482398 | 60.41219343 | 0 | 0 | 1536.402098 |
| LAMB3 | 2.287027903 | 10.20744707 | 44.05098822 | 0 | 0 | 874.4188432 |
| CDC45 | 2.291657161 | 7.128425424 | 67.73467318 | 0 | 0 | 1864.239149 |
| PRSS3 | 2.292207816 | 4.81088655 | 33.27227163 | 8.97E-230 | 1.00E-228 | 514.9454357 |
| ASPM | 2.293043729 | 8.065880981 | 61.69542359 | 0 | 0 | 1592.736875 |
| CYP2S1 | 2.29384877 | 7.474342916 | 44.15279192 | 0 | 0 | 878.1480631 |
| KIF2C | 2.295588569 | 8.285258098 | 68.52278176 | 0 | 0 | 1900.360219 |
| MSLN | 2.297322765 | 6.13569178 | 27.95466274 | 2.32E-165 | 1.49E-164 | 366.8206287 |
| MUC4 | 2.301982039 | 6.03689509 | 32.42083579 | 7.51E-219 | 7.64E-218 | 489.8230181 |
| C1orf106 | 2.302202326 | 8.682863507 | 42.61637868 | 0 | 0 | 822.4806046 |

| | | | | | | |
|---|---|---|---|---|---|---|
| PLK1 | 2.305163276 | 8.499803791 | 69.34528917 | 0 | 0 | 1938.208975 |
| SLPI | 2.30629012 | 9.214143661 | 31.5522218 | 6.10E-208 | 5.63E-207 | 464.7331665 |
| DEPDC1 | 2.307117418 | 7.045304052 | 64.82590568 | 0 | 0 | 1732.238719 |
| IL1A | 2.326118562 | 3.648834682 | 49.40373312 | 0 | 0 | 1077.853869 |
| IL20RB | 2.33164123 | 5.345422682 | 47.3150625 | 0 | 0 | 996.7407965 |
| POF1B | 2.351244241 | 6.069257755 | 34.25057 | 1.34E-242 | 1.65E-241 | 544.4457343 |
| BIRC5 | 2.365133234 | 7.886903282 | 64.41299971 | 0 | 0 | 1713.679437 |
| CENPA | 2.369739905 | 6.406428451 | 67.35731683 | 0 | 0 | 1846.995675 |
| FUT3 | 2.377113992 | 6.035926449 | 37.34766816 | 4.44E-285 | 7.72E-284 | 642.1619962 |
| FAM83D | 2.380493147 | 7.9758651 | 66.82681827 | 0 | 0 | 1822.812474 |
| MELK | 2.395183893 | 7.614327597 | 68.25399211 | 0 | 0 | 1888.024692 |
| GSDMC | 2.398339746 | 4.090913653 | 46.85895959 | 0 | 0 | 979.3150079 |
| GPR87 | 2.410004453 | 4.225711203 | 36.04587171 | 6.97E-267 | 1.04E-265 | 600.304306 |
| EPR1 | 2.414873908 | 7.833206824 | 64.51207447 | 0 | 0 | 1718.128407 |
| CEP55 | 2.457443525 | 7.938530414 | 68.00752643 | 0 | 0 | 1876.728348 |
| RRM2 | 2.47038693 | 9.298832325 | 71.52253062 | 0 | 0 | 2039.098486 |
| GBP6 | 2.503845998 | 3.894451173 | 41.61931184 | 0 | 0 | 787.0732454 |
| ARNTL2 | 2.524916004 | 6.329943247 | 68.1158995 | 0 | 0 | 1881.693707 |
| AURKB | 2.536274715 | 7.424279128 | 68.70038341 | 0 | 0 | 1908.519927 |
| CDC20 | 2.545052516 | 8.807141021 | 69.36730639 | 0 | 0 | 1939.224192 |
| CSTA | 2.548392338 | 6.795014072 | 46.07854204 | 0 | 0 | 949.7448031 |
| SERPINB | 2.554409175 | 3.591440198 | 43.38959069 | 0 | 0 | 850.3304378 |
| CLCA2 | 2.555835573 | 4.458079574 | 31.41828266 | 2.79E-206 | 2.54E-205 | 460.9134196 |
| DLGAP5 | 2.55683467 | 7.470558987 | 66.35688901 | 0 | 0 | 1801.448367 |
| ALDH3A | 2.559180701 | 5.738397719 | 40.60622514 | 0 | 0 | 751.6941184 |
| UBE2C | 2.571233167 | 8.693149425 | 66.49450269 | 0 | 0 | 1807.698899 |
| IL8 | 2.579739977 | 7.377564165 | 50.17420528 | 0 | 0 | 1108.301855 |
| LCN2 | 2.593552873 | 7.973284106 | 30.35424517 | 2.73E-193 | 2.22E-192 | 431.0407242 |
| ANXA8L | 2.624771326 | 4.859813493 | 38.77373959 | 1.46E-305 | 2.98E-304 | 689.2786001 |
| IGF2BP3 | 2.631688154 | 6.022169774 | 53.42628016 | 0 | 0 | 1239.758064 |
| IL1RN | 2.658996422 | 7.662063964 | 53.86343142 | 0 | 0 | 1257.77417 |
| FXYD3 | 2.682580125 | 8.71473933 | 31.32252341 | 4.27E-205 | 3.84E-204 | 458.1906026 |
| GJB2 | 2.690337939 | 8.558064876 | 47.46820263 | 0 | 0 | 1002.615139 |
| TRIM29 | 2.721894781 | 8.164896571 | 35.00239987 | 1.19E-252 | 1.58E-251 | 567.5700227 |
| KRT13 | 2.77051474 | 4.710341036 | 31.04312363 | 1.17E-201 | 1.02E-200 | 450.2848613 |
| CXCL1 | 2.783646591 | 6.456525019 | 47.48051182 | 0 | 0 | 1003.08782 |
| AIM1L | 2.78726418 | 5.131283347 | 55.89814835 | 0 | 0 | 1342.641137 |
| GJB3 | 2.78769782 | 6.147702122 | 44.1043208 | 0 | 0 | 876.3717841 |
| MYBL2 | 2.838731073 | 9.005770089 | 65.25946977 | 0 | 0 | 1751.776037 |
| ANXA8 | 2.862560787 | 5.443022866 | 36.85746437 | 3.60E-278 | 5.99E-277 | 626.269035 |
| S100A2 | 2.978129062 | 7.556530886 | 41.56532432 | 0 | 0 | 785.1725694 |
| PCSK9 | 3.042486681 | 5.25076714 | 47.58433241 | 0 | 0 | 1007.077611 |
| SFN | 3.1107122 | 9.507768401 | 40.99384326 | 0 | 0 | 765.1584964 |
| CEACAM | 3.172967321 | 7.194079107 | 32.5658184 | 1.08E-220 | 1.11E-219 | 494.0641356 |
| PITX1 | 3.176630076 | 7.001275581 | 50.04420873 | 0 | 0 | 1103.145203 |

| | | | | | | |
|---|---|---|---|---|---|---|
| S100A8 | 3.220525255 | 7.344505274 | 49.67546888 | 0 | 0 | 1088.56084 |
| S100P | 3.27326187 | 6.67435326 | 41.4355153 | 0 | 0 | 780.6095429 |
| TMPRSS4 | 3.332892592 | 7.017548391 | 38.94124426 | 5.29E-308 | 1.09E-306 | 694.8976126 |
| KRT17 | 3.358341666 | 8.943816588 | 37.35748657 | 3.22E-285 | 5.62E-284 | 642.4819214 |
| S100A9 | 3.48732839 | 9.03480883 | 57.33653654 | 0 | 0 | 1403.595884 |
| FAM83A | 3.51875714 | 5.160787291 | 49.8084952 | 0 | 0 | 1093.814983 |
| GPX2 | 3.59777086 | 6.4159072 | 40.2104997 | 0 | 0 | 738.041351 |
| PI3 | 4.065204021 | 5.781042386 | 53.91559111 | 0 | 0 | 1259.929049 |
