# Supplementary material for: Multi-omics analysis of lactylation as a prognostic signature: A pan-cancer study
Source: Genes Dis. 2025 Jul 12;13(2):101769. doi: 10.1016/j.gendis.2025.101769 (PMC12664809; doi:10.1016/j.gendis.2025.101769)
Supplement: Multimedia component 14 [file mmc14.pdf]

Supplemental Table S4

ONTOLOGY	ID	Description	GeneRatio	BgRatio	pvalue	p.adjust	qvalue	Count	zscore
BP	GO:0140014	mitotic nuclear division	25/126	293/18800	9.33507E-21	1.87728E-17	1.64297E-17	25	5
BP	GO:0000280	nuclear division	28/126	446/18800	1.34383E-19	1.09245E-16	9.56102E-17	28	5.291503
BP	GO:0048285	organelle fission	29/126	493/18800	1.62972E-19	1.09245E-16	9.56102E-17	29	5.013774
BP	GO:0000070	mitotic sister chromatid segregation	18/126	171/18800	8.76684E-17	4.27903E-14	3.74495E-14	18	4.242641
BP	GO:0007059	chromosome segregation	23/126	348/18800	1.12633E-16	4.27903E-14	3.74495E-14	23	4.795832
BP	GO:0000819	sister chromatid segregation	19/126	205/18800	1.27669E-16	4.27903E-14	3.74495E-14	19	4.358899
BP	GO:0098813	nuclear chromosome segregation	20/126	287/18800	4.85467E-15	1.39468E-12	1.2206E-12	20	4.472136
BP	GO:0044772	mitotic cell cycle phase transition	20/126	440/18800	1.37614E-11	3.45928E-09	3.02751E-09	20	4.472136
BP	GO:1902850	microtubule cytoskeleton organization involved in mitosis	13/126	151/18800	2.69062E-11	6.01205E-09	5.26166E-09	13	3.605551
BP	GO:0007051	spindle organization	14/126	188/18800	3.1872E-11	6.40946E-09	5.60947E-09	14	3.741657
BP	GO:0051783	regulation of nuclear division	12/126	139/18800	1.56658E-10	2.86398E-08	2.50652E-08	12	3.464102
BP	GO:0007088	regulation of mitotic nuclear division	11/126	111/18800	2.11432E-10	3.54324E-08	3.101E-08	11	3.316625
BP	GO:0007052	mitotic spindle organization	11/126	124/18800	7.02216E-10	1.08627E-07	9.50692E-08	11	3.316625
BP	GO:0000910	cytokinesis	12/126	174/18800	2.1061E-09	3.02526E-07	2.64767E-07	12	3.464102
BP	GO:0051321	meiotic cell cycle	14/126	266/18800	3.07527E-09	4.01628E-07	3.51499E-07	14	3.741657
BP	GO:0007346	regulation of mitotic cell cycle	18/126	478/18800	3.19545E-09	4.01628E-07	3.51499E-07	18	4.242641
BP	GO:0051302	regulation of cell division	12/126	186/18800	4.49583E-09	5.3183E-07	4.6545E-07	12	3.464102
BP	GO:0051225	spindle assembly	10/126	121/18800	8.60743E-09	9.61642E-07	8.41616E-07	10	3.162278
BP	GO:0051983	regulation of chromosome segregation	9/126	91/18800	1.00907E-08	1.06802E-06	9.34717E-07	9	3
BP	GO:0090068	positive regulation of cell cycle process	13/126	247/18800	1.1619E-08	1.16829E-06	1.02248E-06	13	3.605551
BP	GO:1903046	meiotic cell cycle process	12/126	207/18800	1.497E-08	1.43356E-06	1.25463E-06	12	3.464102
BP	GO:0051304	chromosome separation	9/126	97/18800	1.77902E-08	1.57124E-06	1.37513E-06	9	3
BP	GO:1901987	regulation of cell cycle phase transition	16/126	415/18800	1.85411E-08	1.57124E-06	1.37513E-06	16	4
BP	GO:0090307	mitotic spindle assembly	8/126	69/18800	1.93184E-08	1.57124E-06	1.37513E-06	8	2.828427
BP	GO:0033044	regulation of chromosome organization	12/126	212/18800	1.95331E-08	1.57124E-06	1.37513E-06	12	2.886751
BP	GO:0061640	cytoskeleton-dependent cytokinesis	9/126	100/18800	2.32838E-08	1.80091E-06	1.57613E-06	9	3
BP	GO:0000281	mitotic cytokinesis	8/126	71/18800	2.43076E-08	1.81047E-06	1.58449E-06	8	2.828427
BP	GO:1901990	regulation of mitotic cell cycle phase transition	14/126	321/18800	3.33252E-08	2.39346E-06	2.09472E-06	14	3.741657
BP	GO:0140694	non-membrane-bounded organelle assembly	15/126	379/18800	3.78262E-08	2.62305E-06	2.29566E-06	15	3.356586
BP	GO:0045787	positive regulation of cell cycle	14/126	329/18800	4.52903E-08	3.03596E-06	2.65703E-06	14	3.741657
BP	GO:0140013	meiotic nuclear division	11/126	188/18800	5.58276E-08	3.62159E-06	3.16957E-06	11	3.316625
BP	GO:0007143	female meiotic nuclear division	6/126	33/18800	7.67767E-08	4.82494E-06	4.22272E-06	6	2.44949
BP	GO:0044839	cell cycle G2/M phase transition	10/126	155/18800	9.21336E-08	5.61457E-06	4.91379E-06	10	3.162278
BP	GO:0030071	regulation of mitotic metaphase/anaphase transition	7/126	60/18800	1.47703E-07	8.73623E-06	7.64583E-06	7	2.645751
BP	GO:0007091	metaphase/anaphase transition of mitotic cell cycle	7/126	62/18800	1.86012E-07	1.06877E-05	9.35375E-06	7	2.645751
BP	GO:1902099	regulation of metaphase/anaphase transition of cell cycle	7/126	63/18800	2.08107E-07	1.16251E-05	1.01741E-05	7	2.645751
BP	GO:0010965	regulation of mitotic sister chromatid separation	7/126	65/18800	2.58978E-07	1.37054E-05	1.19948E-05	7	2.645751
BP	GO:0044784	metaphase/anaphase transition of cell cycle	7/126	65/18800	2.58978E-07	1.37054E-05	1.19948E-05	7	2.645751

BP	GO:0051306	mitotic sister chromatid separation	7/126	68/18800	3.54681E-07	1.82888E-05	1.60061E-05	7	2.645751
BP	GO:0000086	G2/M transition of mitotic cell cycle	9/126	140/18800	4.28234E-07	2.15295E-05	1.88423E-05	9	3
BP	GO:0033045	regulation of sister chromatid segregation	7/126	72/18800	5.27139E-07	2.52399E-05	2.20896E-05	7	2.645751
BP	GO:1905818	regulation of chromosome separation	7/126	72/18800	5.27139E-07	2.52399E-05	2.20896E-05	7	2.645751
BP	GO:0051303	establishment of chromosome localization	7/126	81/18800	1.18378E-06	5.53626E-05	4.84526E-05	7	2.645751
BP	GO:0050000	chromosome localization	7/126	83/18800	1.39791E-06	6.3891E-05	5.59165E-05	7	2.645751
BP	GO:0032465	regulation of cytokinesis	7/126	91/18800	2.60711E-06	0.000116509	0.000101967	7	2.645751
BP	GO:0045132	meiotic chromosome segregation	7/126	96/18800	3.73445E-06	0.000163261	0.000142883	7	2.645751
BP	GO:0051310	metaphase plate congression	6/126	65/18800	4.80615E-06	0.000205642	0.000179975	6	2.44949
BP	GO:0051782	negative regulation of cell division	4/126	18/18800	5.47291E-06	0.000229292	0.000200673	4	2
BP	GO:0010948	negative regulation of cell cycle process	11/126	301/18800	5.86265E-06	0.000240608	0.000210577	11	3.316625
BP	GO:0007292	female gamete generation	8/126	158/18800	1.13104E-05	0.000454906	0.000398128	8	2.828427
BP	GO:0030261	chromosome condensation	5/126	49/18800	1.88012E-05	0.000741358	0.000648827	5	2.236068
BP	GO:0051307	meiotic chromosome separation	4/126	25/18800	2.18176E-05	0.000843755	0.000738443	4	2
BP	GO:0007080	mitotic metaphase plate congression	5/126	51/18800	2.29143E-05	0.000869448	0.000760929	5	2.236068
BP	GO:2001251	negative regulation of chromosome organization	6/126	89/18800	2.96602E-05	0.001088358	0.000952516	6	2.44949
BP	GO:0052548	regulation of endopeptidase activity	12/126	426/18800	2.97661E-05	0.001088358	0.000952516	12	2.886751
BP	GO:0051231	spindle elongation	3/126	10/18800	3.40828E-05	0.001223939	0.001071175	3	1.732051
BP	GO:1904668	positive regulation of ubiquitin protein ligase activity	3/126	11/18800	4.66344E-05	0.001645293	0.001439938	3	1.732051
BP	GO:0010389	regulation of G2/M transition of mitotic cell cycle	6/126	97/18800	4.82844E-05	0.001674138	0.001465183	6	2.44949
BP	GO:0000079	regulation of cyclin-dependent protein serine/threonine kir	6/126	98/18800	5.11539E-05	0.001743566	0.001525945	6	2.44949
BP	GO:0034508	centromere complex assembly	4/126	31/18800	5.2606E-05	0.001763178	0.001543109	4	2
BP	GO:0052547	regulation of peptidase activity	12/126	456/18800	5.7469E-05	0.001894593	0.001658122	12	2.886751
BP	GO:0045786	negative regulation of cell cycle	11/126	387/18800	5.97508E-05	0.001914308	0.001675377	11	3.316625
BP	GO:1904029	regulation of cyclin-dependent protein kinase activity	6/126	101/18800	6.05899E-05	0.001914308	0.001675377	6	2.44949
BP	GO:0044771	meiotic cell cycle phase transition	3/126	12/18800	6.18747E-05	0.001914308	0.001675377	3	1.732051
BP	GO:0051255	spindle midzone assembly	3/126	12/18800	6.18747E-05	0.001914308	0.001675377	3	1.732051
BP	GO:0051656	establishment of organelle localization	11/126	404/18800	8.76586E-05	0.002643169	0.002313266	11	2.713602
BP	GO:1902749	regulation of cell cycle G2/M phase transition	6/126	108/18800	8.80618E-05	0.002643169	0.002313266	6	2.44949
BP	GO:0008608	attachment of spindle microtubules to kinetochore	4/126	37/18800	0.000107042	0.003165599	0.002770489	4	2
BP	GO:0007094	mitotic spindle assembly checkpoint signaling	4/126	38/18800	0.000119017	0.003371037	0.002950286	4	2
BP	GO:0071173	spindle assembly checkpoint signaling	4/126	38/18800	0.000119017	0.003371037	0.002950286	4	2
BP	GO:0071174	mitotic spindle checkpoint signaling	4/126	38/18800	0.000119017	0.003371037	0.002950286	4	2
BP	GO:0000212	meiotic spindle organization	3/126	15/18800	0.000126099	0.003426816	0.002999103	3	1.732051
BP	GO:0051315	attachment of mitotic spindle microtubules to kinetochore	3/126	15/18800	0.000126099	0.003426816	0.002999103	3	1.732051
BP	GO:0060707	trophoblast giant cell differentiation	3/126	15/18800	0.000126099	0.003426816	0.002999103	3	1.732051
BP	GO:0031577	spindle checkpoint signaling	4/126	39/18800	0.000131935	0.003491062	0.00305533	4	2
BP	GO:0032506	cytokinetic process	4/126	39/18800	0.000131935	0.003491062	0.00305533	4	2
BP	GO:0045841	negative regulation of mitotic metaphase/anaphase transit	4/126	40/18800	0.000145838	0.003808825	0.003333432	4	2
BP	GO:0000075	cell cycle checkpoint signaling	7/126	170/18800	0.000149579	0.003856443	0.003375107	7	2.645751

BP	GO:0051238	sequestering of metal ion	3/126	16/18800	0.000154439	0.00393136	0.003440673	3	1.732051
BP	GO:1902100	negative regulation of metaphase/anaphase transition of c	4/126	42/18800	0.000176777	0.004443724	0.003889087	4	2
BP	GO:1901989	positive regulation of cell cycle phase transition	6/126	123/18800	0.000180228	0.004474545	0.003916061	6	2.44949
BP	GO:0033046	negative regulation of sister chromatid segregation	4/126	43/18800	0.000193902	0.004642117	0.004062718	4	2
BP	GO:0033048	negative regulation of mitotic sister chromatid segregation	4/126	43/18800	0.000193902	0.004642117	0.004062718	4	2
BP	GO:2000816	negative regulation of mitotic sister chromatid separation	4/126	43/18800	0.000193902	0.004642117	0.004062718	4	2
BP	GO:0045840	positive regulation of mitotic nuclear division	4/126	44/18800	0.000212193	0.005020233	0.00439364	4	2
BP	GO:0031145	anaphase-promoting complex-dependent catabolic proce	3/126	18/18800	0.000222845	0.005210952	0.004560555	3	1.732051
BP	GO:0051985	negative regulation of chromosome segregation	4/126	45/18800	0.000231694	0.005294733	0.004633879	4	2
BP	GO:1905819	negative regulation of chromosome separation	4/126	45/18800	0.000231694	0.005294733	0.004633879	4	2
BP	GO:1901991	negative regulation of mitotic cell cycle phase transition	7/126	183/18800	0.000235338	0.005317576	0.004653871	7	2.645751
BP	GO:0007093	mitotic cell cycle checkpoint signaling	6/126	130/18800	0.000243478	0.005440378	0.004761345	6	2.44949
BP	GO:0033047	regulation of mitotic sister chromatid segregation	4/126	46/18800	0.000252452	0.005578912	0.004882589	4	2
BP	GO:0007076	mitotic chromosome condensation	3/126	19/18800	0.000263336	0.005756175	0.005037726	3	1.732051
BP	GO:0010951	negative regulation of endopeptidase activity	8/126	251/18800	0.000292534	0.006325665	0.005536137	8	2.828427
BP	GO:0045839	negative regulation of mitotic nuclear division	4/126	48/18800	0.000297927	0.006373729	0.005578201	4	2
BP	GO:1901988	negative regulation of cell cycle phase transition	8/126	255/18800	0.000325266	0.006885365	0.006025978	8	2.828427
BP	GO:0051781	positive regulation of cell division	5/126	89/18800	0.000330542	0.006924162	0.006059933	5	2.236068
BP	GO:0051445	regulation of meiotic cell cycle	4/126	51/18800	0.000376747	0.007810697	0.006835816	4	2
BP	GO:0010466	negative regulation of peptidase activity	8/126	262/18800	0.000389718	0.007997183	0.006999026	8	2.828427
BP	GO:0001890	placenta development	6/126	147/18800	0.000470498	0.009491034	0.008306424	6	2.44949
BP	GO:0051383	kinetochore organization	3/126	23/18800	0.000471956	0.009491034	0.008306424	3	1.732051
BP	GO:0051653	spindle localization	4/126	55/18800	0.000503666	0.009930126	0.008690712	4	2
BP	GO:0051784	negative regulation of nuclear division	4/126	55/18800	0.000503666	0.009930126	0.008690712	4	2
BP	GO:1904666	regulation of ubiquitin protein ligase activity	3/126	24/18800	0.000536746	0.010479584	0.00917159	3	1.732051
BP	GO:1901992	positive regulation of mitotic cell cycle phase transition	5/126	100/18800	0.000565448	0.010933805	0.009569118	5	2.236068
BP	GO:0006260	DNA replication	8/126	280/18800	0.000604332	0.011515139	0.010077893	8	2.828427
BP	GO:0071459	protein localization to chromosome, centromeric region	3/126	25/18800	0.000606964	0.011515139	0.010077893	3	1.732051
BP	GO:0051785	positive regulation of nuclear division	4/126	59/18800	0.000658404	0.012374295	0.010829816	4	2
BP	GO:0048608	reproductive structure development	10/126	433/18800	0.000677541	0.012597116	0.011024825	10	2.529822
BP	GO:0060706	cell differentiation involved in embryonic placenta develop	3/126	26/18800	0.000682787	0.012597116	0.011024825	3	1.732051
BP	GO:0061458	reproductive system development	10/126	436/18800	0.000714387	0.013060296	0.011430195	10	2.529822
BP	GO:0010972	negative regulation of G2/M transition of mitotic cell cycle	4/126	62/18800	0.000794621	0.014396245	0.012599399	4	2
BP	GO:0030307	positive regulation of cell growth	6/126	163/18800	0.000810123	0.014546048	0.012730505	6	0.816497
BP	GO:0007062	sister chromatid cohesion	4/126	63/18800	0.000844134	0.015022603	0.013147579	4	2
BP	GO:0035116	embryonic hindlimb morphogenesis	3/126	28/18800	0.000851946	0.015028628	0.013152852	3	-0.57735
BP	GO:1902750	negative regulation of cell cycle G2/M phase transition	4/126	64/18800	0.000895785	0.015664546	0.013709399	4	2
BP	GO:0045930	negative regulation of mitotic cell cycle	7/126	234/18800	0.001019484	0.017673992	0.015468039	7	2.645751
BP	GO:0051443	positive regulation of ubiquitin-protein transferase activity	3/126	32/18800	0.001264939	0.021557564	0.018866888	3	1.732051
BP	GO:0071711	basement membrane organization	3/126	32/18800	0.001264939	0.021557564	0.018866888	3	0.57735

BP	GO:0051052	regulation of DNA metabolic process	10/126	472/18800	0.001301259	0.02199019	0.019245517	10	3.162278
BP	GO:0019730	antimicrobial humoral response	5/126	122/18800	0.001385148	0.023212765	0.020315498	5	2.236068
BP	GO:0031109	microtubule polymerization or depolymerization	5/126	123/18800	0.001436204	0.023869465	0.020890233	5	1.341641
BP	GO:0051973	positive regulation of telomerase activity	3/126	34/18800	0.001511274	0.024911246	0.021801985	3	1.732051
BP	GO:0007095	mitotic G2 DNA damage checkpoint signaling	3/126	35/18800	0.001644917	0.026455249	0.023153276	3	1.732051
BP	GO:0035137	hindlimb morphogenesis	3/126	35/18800	0.001644917	0.026455249	0.023153276	3	-0.57735
BP	GO:0040001	establishment of mitotic spindle localization	3/126	35/18800	0.001644917	0.026455249	0.023153276	3	1.732051
BP	GO:0022412	cellular process involved in reproduction in multicellular or	9/126	406/18800	0.001668762	0.026455249	0.023153276	9	2.333333
BP	GO:0044843	cell cycle G1/S phase transition	7/126	255/18800	0.001670719	0.026455249	0.023153276	7	2.645751
BP	GO:0045931	positive regulation of mitotic cell cycle	5/126	128/18800	0.001712546	0.026905703	0.023547508	5	2.236068
BP	GO:1901993	regulation of meiotic cell cycle phase transition	2/126	10/18800	0.001936045	0.029949125	0.026211069	2	1.414214
BP	GO:1903867	extraembryonic membrane development	2/126	10/18800	0.001936045	0.029949125	0.026211069	2	1.414214
BP	GO:0051054	positive regulation of DNA metabolic process	7/126	263/18800	0.001990147	0.03055104	0.026737857	7	2.645751
BP	GO:0061982	meiosis I cell cycle process	5/126	136/18800	0.002233202	0.034022487	0.029776021	5	2.236068
BP	GO:0035404	histone-serine phosphorylation	2/126	11/18800	0.002355914	0.03535629	0.030943347	2	1.414214
BP	GO:0060623	regulation of chromosome condensation	2/126	11/18800	0.002355914	0.03535629	0.030943347	2	1.414214
BP	GO:0045740	positive regulation of DNA replication	3/126	40/18800	0.002423344	0.036098846	0.031593222	3	1.732051
BP	GO:0045861	negative regulation of proteolysis	8/126	350/18800	0.002495112	0.036894631	0.032289682	8	2.828427
BP	GO:0008544	epidermis development	8/126	355/18800	0.002721898	0.039954277	0.034967442	8	2.828427
BP	GO:0010639	negative regulation of organelle organization	8/126	356/18800	0.002769131	0.04034328	0.035307893	8	2.12132
BP	GO:0032467	positive regulation of cytokinesis	3/126	42/18800	0.002788521	0.04034328	0.035307893	3	1.732051
BP	GO:0014002	astrocyte development	3/126	43/18800	0.002983118	0.042546449	0.037236077	3	0.57735
BP	GO:0044786	cell cycle DNA replication	3/126	43/18800	0.002983118	0.042546449	0.037236077	3	1.732051
BP	GO:2001252	positive regulation of chromosome organization	4/126	89/18800	0.00302745	0.042874667	0.037523329	4	2
BP	GO:0031589	cell-substrate adhesion	8/126	364/18800	0.003170506	0.044586631	0.039021616	8	2.828427
BP	GO:0051988	regulation of attachment of spindle microtubules to kineto	2/126	13/18800	0.003311936	0.045933126	0.040200051	2	1.414214
BP	GO:0070486	leukocyte aggregation	2/126	13/18800	0.003311936	0.045933126	0.040200051	2	1.414214
BP	GO:0016572	histone phosphorylation	3/126	45/18800	0.003396881	0.04678854	0.040948697	3	1.732051
BP	GO:0034502	protein localization to chromosome	4/126	93/18800	0.003546853	0.048341102	0.042307479	4	2
BP	GO:0051346	negative regulation of hydrolase activity	8/126	371/18800	0.003557674	0.048341102	0.042307479	8	2.828427
BP	GO:0007019	microtubule depolymerization	3/126	46/18800	0.003616259	0.048807365	0.042715546	3	1.732051
BP	GO:0097530	granulocyte migration	5/126	154/18800	0.003813473	0.049276283	0.043125936	5	2.236068
BP	GO:0021700	developmental maturation	7/126	296/18800	0.003835037	0.049276283	0.043125936	7	1.133893
BP	GO:0043588	skin development	7/126	296/18800	0.003835037	0.049276283	0.043125936	7	2.645751
BP	GO:0043616	keratinocyte proliferation	3/126	47/18800	0.003844106	0.049276283	0.043125936	3	1.732051
BP	GO:0051972	regulation of telomerase activity	3/126	47/18800	0.003844106	0.049276283	0.043125936	3	1.732051
BP	GO:0014733	regulation of skeletal muscle adaptation	2/126	14/18800	0.00384703	0.049276283	0.043125936	2	0
BP	GO:0090128	regulation of synapse maturation	2/126	14/18800	0.00384703	0.049276283	0.043125936	2	0
BP	GO:2000105	positive regulation of DNA-templated DNA replication	2/126	14/18800	0.00384703	0.049276283	0.043125936	2	1.414214
BP	GO:0046777	protein autophosphorylation	6/126	224/18800	0.004026004	0.050949304	0.044590142	6	1.632993

BP	GO:0007605	sensory perception of sound	5/126	156/18800	0.004028314	0.050949304	0.044590142	5	0.447214
BP	GO:0044818	mitotic G2/M transition checkpoint	3/126	48/18800	0.004080517	0.051287004	0.044885692	3	1.732051
BP	GO:0006261	DNA-templated DNA replication	5/126	159/18800	0.004366623	0.053864092	0.047141125	5	2.236068
BP	GO:0016446	somatic hypermutation of immunoglobulin genes	2/126	15/18800	0.00441948	0.053864092	0.047141125	2	1.414214
BP	GO:0045842	positive regulation of mitotic metaphase/anaphase transiti	2/126	15/18800	0.00441948	0.053864092	0.047141125	2	1.414214
BP	GO:0070365	hepatocyte differentiation	2/126	15/18800	0.00441948	0.053864092	0.047141125	2	1.414214
BP	GO:1901970	positive regulation of mitotic sister chromatid separation	2/126	15/18800	0.00441948	0.053864092	0.047141125	2	1.414214
BP	GO:0097529	myeloid leukocyte migration	6/126	229/18800	0.004480753	0.05395685	0.047222305	6	2.44949
BP	GO:2000116	regulation of cysteine-type endopeptidase activity	6/126	229/18800	0.004480753	0.05395685	0.047222305	6	1.632993
BP	GO:0051293	establishment of spindle localization	3/126	50/18800	0.004579415	0.054504068	0.047701223	3	1.732051
BP	GO:0001701	in utero embryonic development	8/126	387/18800	0.004580402	0.054504068	0.047701223	8	2.828427
BP	GO:0002566	somatic diversification of immune receptors via somatic m	2/126	16/18800	0.005028772	0.058455841	0.051159762	2	1.414214
BP	GO:0017014	protein nitrosylation	2/126	16/18800	0.005028772	0.058455841	0.051159762	2	1.414214
BP	GO:0018119	peptidyl-cysteine S-nitrosylation	2/126	16/18800	0.005028772	0.058455841	0.051159762	2	1.414214
BP	GO:1902101	positive regulation of metaphase/anaphase transition of ce	2/126	16/18800	0.005028772	0.058455841	0.051159762	2	1.414214
BP	GO:0007160	cell-matrix adhesion	6/126	235/18800	0.005075272	0.058643958	0.051324399	6	2.44949
BP	GO:0051100	negative regulation of binding	5/126	165/18800	0.005103278	0.058643958	0.051324399	5	2.236068
BP	GO:0007018	microtubule-based movement	8/126	395/18800	0.005169932	0.059072343	0.051699316	8	2.12132
BP	GO:0030216	keratinocyte differentiation	5/126	167/18800	0.005367286	0.060980861	0.053369625	5	2.236068
BP	GO:0010638	positive regulation of organelle organization	9/126	487/18800	0.005550025	0.062017412	0.0542768	9	2.333333
BP	GO:0000076	DNA replication checkpoint signaling	2/126	17/18800	0.005674393	0.062017412	0.0542768	2	1.414214
BP	GO:0002544	chronic inflammatory response	2/126	17/18800	0.005674393	0.062017412	0.0542768	2	1.414214
BP	GO:0016264	gap junction assembly	2/126	17/18800	0.005674393	0.062017412	0.0542768	2	1.414214
BP	GO:0033623	regulation of integrin activation	2/126	17/18800	0.005674393	0.062017412	0.0542768	2	1.414214
BP	GO:0061952	midbody abscission	2/126	17/18800	0.005674393	0.062017412	0.0542768	2	1.414214
BP	GO:1904355	positive regulation of telomere capping	2/126	17/18800	0.005674393	0.062017412	0.0542768	2	1.414214
BP	GO:0051438	regulation of ubiquitin-protein transferase activity	3/126	56/18800	0.006290948	0.06739522	0.058983385	3	1.732051
BP	GO:0007096	regulation of exit from mitosis	2/126	18/18800	0.006355836	0.06739522	0.058983385	2	1.414214
BP	GO:0008356	asymmetric cell division	2/126	18/18800	0.006355836	0.06739522	0.058983385	2	0
BP	GO:0051382	kinetochore assembly	2/126	18/18800	0.006355836	0.06739522	0.058983385	2	1.414214
BP	GO:1905820	positive regulation of chromosome separation	2/126	18/18800	0.006355836	0.06739522	0.058983385	2	1.414214
BP	GO:0042770	signal transduction in response to DNA damage	5/126	174/18800	0.006367525	0.06739522	0.058983385	5	2.236068
BP	GO:2000241	regulation of reproductive process	5/126	176/18800	0.006675814	0.070288282	0.061515353	5	2.236068
BP	GO:0050954	sensory perception of mechanical stimulus	5/126	177/18800	0.00683382	0.071577141	0.062643346	5	0.447214
BP	GO:0001558	regulation of cell growth	8/126	415/18800	0.006898508	0.071833336	0.062867564	8	1.414214
BP	GO:0002523	leukocyte migration involved in inflammatory response	2/126	19/18800	0.007072601	0.071833336	0.062867564	2	1.414214
BP	GO:0034501	protein localization to kinetochore	2/126	19/18800	0.007072601	0.071833336	0.062867564	2	1.414214
BP	GO:0045091	regulation of single stranded viral RNA replication via doub	2/126	19/18800	0.007072601	0.071833336	0.062867564	2	1.414214
BP	GO:0051797	regulation of hair follicle development	2/126	19/18800	0.007072601	0.071833336	0.062867564	2	1.414214
BP	GO:1903083	protein localization to condensed chromosome	2/126	19/18800	0.007072601	0.071833336	0.062867564	2	1.414214

BP	GO:0032496	response to lipopolysaccharide	7/126	333/18800	0.007205301	0.072813373	0.06372528	7	2.645751
BP	GO:0045927	positive regulation of growth	6/126	256/18800	0.007617081	0.076589745	0.067030309	6	0.816497
BP	GO:0039692	single stranded viral RNA replication via double stranded	2/126	20/18800	0.007824191	0.078280833	0.068510326	2	1.414214
BP	GO:0030326	embryonic limb morphogenesis	4/126	119/18800	0.008446981	0.083679203	0.073234906	4	0
BP	GO:0035113	embryonic appendage morphogenesis	4/126	119/18800	0.008446981	0.083679203	0.073234906	4	0
BP	GO:0007063	regulation of sister chromatid cohesion	2/126	21/18800	0.008610114	0.084463118	0.073920979	2	1.414214
BP	GO:0033762	response to glucagon	2/126	21/18800	0.008610114	0.084463118	0.073920979	2	1.414214
BP	GO:0044321	response to leptin	2/126	22/18800	0.009429884	0.091488431	0.080069437	2	0
BP	GO:0070269	pyroptosis	2/126	22/18800	0.009429884	0.091488431	0.080069437	2	1.414214
BP	GO:0031570	DNA integrity checkpoint signaling	4/126	123/18800	0.009462752	0.091488431	0.080069437	4	2
BP	GO:0002237	response to molecule of bacterial origin	7/126	354/18800	0.009898728	0.095245654	0.083357708	7	2.645751
BP	GO:1902410	mitotic cytokinetic process	2/126	23/18800	0.010283021	0.098472163	0.086181505	2	1.414214
CC	GO:0005819	spindle	20/129	402/19594	1.99732E-12	4.27426E-10	3.57414E-10	20	4.472136
CC	GO:0000775	chromosome, centromeric region	15/129	227/19594	2.66106E-11	2.3643E-09	1.97703E-09	15	3.872983
CC	GO:0000779	condensed chromosome, centromeric region	13/129	156/19594	3.31443E-11	2.3643E-09	1.97703E-09	13	3.605551
CC	GO:0000793	condensed chromosome	15/129	255/19594	1.37665E-10	7.36507E-09	6.15869E-09	15	3.872983
CC	GO:0000776	kinetochore	12/129	146/19594	2.30156E-10	9.85068E-09	8.23717E-09	12	3.464102
CC	GO:0030496	midbody	12/129	203/19594	1.00072E-08	3.56924E-07	2.98461E-07	12	3.464102
CC	GO:0098687	chromosomal region	15/129	366/19594	1.91966E-08	5.86866E-07	4.90739E-07	15	3.872983
CC	GO:0072686	mitotic spindle	10/129	160/19594	1.06242E-07	2.84198E-06	2.37647E-06	10	3.162278
CC	GO:0005922	connexin complex	5/129	21/19594	2.13949E-07	5.08724E-06	4.25397E-06	5	2.236068
CC	GO:0005874	microtubule	14/129	435/19594	1.09685E-06	2.34727E-05	1.96279E-05	14	3.207135
CC	GO:0000922	spindle pole	9/129	169/19594	1.80429E-06	3.51017E-05	2.93522E-05	9	3
CC	GO:0005921	gap junction	5/129	32/19594	1.99787E-06	3.56287E-05	2.97928E-05	5	2.236068
CC	GO:0051233	spindle midzone	5/129	36/19594	3.66212E-06	6.02842E-05	5.04098E-05	5	2.236068
CC	GO:0005875	microtubule associated complex	8/129	160/19594	1.09563E-05	0.000167475	0.000140043	8	2.828427
CC	GO:0005871	kinesin complex	5/129	49/19594	1.72977E-05	0.000241584	0.000202013	5	2.236068
CC	GO:0005876	spindle microtubule	6/129	83/19594	1.80623E-05	0.000241584	0.000202013	6	2.44949
CC	GO:0097431	mitotic spindle pole	4/129	36/19594	8.9709E-05	0.001129278	0.000944305	4	2
CC	GO:0045171	intercellular bridge	5/129	75/19594	0.000136568	0.001623645	0.001357697	5	2.236068
CC	GO:0000228	nuclear chromosome	7/129	228/19594	0.000792275	0.008923523	0.007461874	7	2.645751
CC	GO:1990752	microtubule end	3/129	33/19594	0.001316553	0.014087122	0.011779689	3	1.732051
CC	GO:0090543	Flemming body	3/129	35/19594	0.001564267	0.015940627	0.013329595	3	1.732051
CC	GO:0000940	outer kinetochore	2/129	12/19594	0.002718653	0.026445081	0.022113447	2	1.414214
CC	GO:0031616	spindle pole centrosome	2/129	14/19594	0.00371629	0.033136919	0.02770918	2	1.414214
CC	GO:1990023	mitotic spindle midzone	2/129	14/19594	0.00371629	0.033136919	0.02770918	2	1.414214
CC	GO:0010369	chromocenter	2/129	15/19594	0.004269609	0.036547854	0.030561413	2	1.414214
CC	GO:0005911	cell-cell junction	9/129	497/19594	0.00565838	0.046572819	0.038944315	9	2.333333
CC	GO:0035580	specific granule lumen	3/129	62/19594	0.007946395	0.062982542	0.052666169	3	1.732051
CC	GO:0005680	anaphase-promoting complex	2/129	21/19594	0.008321891	0.063603027	0.05318502	2	1.414214

CC	GO:0035371	microtubule plus-end	2/129	23/19594	0.009940289	0.073352474	0.061337534	2	1.414214
CC	GO:0032153	cell division site	3/129	73/19594	0.012403647	0.087023147	0.072768986	3	1.732051
CC	GO:0031528	microvillus membrane	2/129	26/19594	0.012606157	0.087023147	0.072768986	2	1.414214
MF	GO:0008017	microtubule binding	16/128	272/18410	7.05283E-11	2.28512E-08	1.98964E-08	16	3.5
MF	GO:0015631	tubulin binding	16/128	376/18410	7.78294E-09	1.26084E-06	1.0978E-06	16	3.5
MF	GO:0005243	gap junction channel activity	5/128	21/18410	2.79546E-07	3.01909E-05	2.62871E-05	5	2.236068
MF	GO:0022829	wide pore channel activity	5/128	30/18410	1.86194E-06	0.000150817	0.000131316	5	2.236068
MF	GO:0003777	microtubule motor activity	6/128	67/18410	7.08184E-06	0.000458903	0.000399565	6	2.44949
MF	GO:0003774	cytoskeletal motor activity	6/128	111/18410	0.000125249	0.00676345	0.005888904	6	2.44949
MF	GO:0004866	endopeptidase inhibitor activity	6/128	180/18410	0.001628573	0.067425844	0.058707363	6	2.44949
MF	GO:0030414	peptidase inhibitor activity	6/128	187/18410	0.001974902	0.067425844	0.058707363	6	2.44949
MF	GO:0000217	DNA secondary structure binding	3/128	36/18410	0.001982288	0.067425844	0.058707363	3	0.57735
MF	GO:0050786	RAGE receptor binding	2/128	10/18410	0.002081045	0.067425844	0.058707363	2	1.414214
MF	GO:0061135	endopeptidase regulator activity	6/128	194/18410	0.002374334	0.06993492	0.060892003	6	2.44949
MF	GO:0035325	Toll-like receptor binding	2/128	12/18410	0.003024533	0.07925778	0.069009373	2	1.414214
MF	GO:0048306	calcium-dependent protein binding	4/128	87/18410	0.003180096	0.07925778	0.069009373	4	2
MF	GO:0003779	actin binding	9/128	439/18410	0.00360147	0.083348305	0.072570974	9	1.666667
MF	GO:0008499	UDP-galactose:beta-N-acetylglucosamine beta-1,3-galact	2/128	14/18410	0.004132429	0.085822825	0.074725526	2	0
MF	GO:0036041	long-chain fatty acid binding	2/128	15/18410	0.004746569	0.085822825	0.074725526	2	1.414214
MF	GO:0004867	serine-type endopeptidase inhibitor activity	4/128	98/18410	0.004867916	0.085822825	0.074725526	4	2
MF	GO:0003688	DNA replication origin binding	2/128	16/18410	0.005400069	0.085822825	0.074725526	2	1.414214
MF	GO:0061134	peptidase regulator activity	6/128	230/18410	0.005457366	0.085822825	0.074725526	6	2.44949
MF	GO:0005149	interleukin-1 receptor binding	2/128	17/18410	0.006092361	0.085822825	0.074725526	2	1.414214
MF	GO:0008301	DNA binding, bending	2/128	17/18410	0.006092361	0.085822825	0.074725526	2	1.414214
MF	GO:0008574	plus-end-directed microtubule motor activity	2/128	17/18410	0.006092361	0.085822825	0.074725526	2	1.414214
MF	GO:0048531	beta-1,3-galactosyltransferase activity	2/128	17/18410	0.006092361	0.085822825	0.074725526	2	0
MF	GO:0034185	apolipoprotein binding	2/128	18/18410	0.006822882	0.090718955	0.078988564	2	0
MF	GO:0015267	channel activity	9/128	489/18410	0.007187469	0.090718955	0.078988564	9	1.666667
MF	GO:0022803	passive transmembrane transporter activity	9/128	490/18410	0.007279916	0.090718955	0.078988564	9	1.666667
KEGG	hsa04110	Cell cycle	10/56	126/8164	1.04568E-08	1.30711E-06	1.27684E-06	10	3.162278
KEGG	hsa04114	Oocyte meiosis	7/56	131/8164	2.81228E-05	0.001757677	0.001716973	7	1.889822
KEGG	hsa04657	IL-17 signaling pathway	5/56	94/8164	0.000436851	0.018202111	0.017780589	5	2.236068
KEGG	hsa04914	Progesterone-mediated oocyte maturation	5/56	102/8164	0.000635888	0.019871507	0.019411324	5	2.236068
