# Supplementary material for: Multi-omics analysis of lactylation as a prognostic signature: A pan-cancer study
Source: Genes Dis. 2025 Jul 12;13(2):101769. doi: 10.1016/j.gendis.2025.101769 (PMC12664809; doi:10.1016/j.gendis.2025.101769)
Supplement: Multimedia component 15 [file mmc15.pdf]

Supplemental Table S5

ID	Description	setSize	enrichmentScore	NES	pvalue	p.adjust	qvalue	rank	leading_edge
REACTOME_CELL_CYCLE	REACTOME_CELL_CYCLE	95	0.559466264	3.864226207	1E-10	2.17273E-09	1.0622E-09	425	tags=82%, list=37%, signal=57%
REACTOME_CELL_CYCLE_MITOTIC	REACTOME_CELL_CYCLE_MITOTIC	79	0.564353049	3.706880051	1E-10	2.17273E-09	1.0622E-09	222	tags=62%, list=19%, signal=54%
REACTOME_CELL_CYCLE_CHECKPOINTS	REACTOME_CELL_CYCLE_CHECKPOINTS	49	0.614684969	3.589528631	1E-10	2.17273E-09	1.0622E-09	306	tags=78%, list=26%, signal=60%
REACTOME_M_PHASE	REACTOME_M_PHASE	43	0.635200951	3.573268209	1E-10	2.17273E-09	1.0622E-09	344	tags=86%, list=30%, signal=63%
REACTOME_RHO_GTPASE_EFFECTORS	REACTOME_RHO_GTPASE_EFFECTORS	37	0.646602033	3.466083187	1E-10	2.17273E-09	1.0622E-09	306	tags=84%, list=26%, signal=64%
REACTOME_MITOTIC_METAPHASE_AND_ANAPHASE	REACTOME_MITOTIC_METAPHASE_AND_ANAPHASE	36	0.651544239	3.430944143	1E-10	2.17273E-09	1.0622E-09	344	tags=89%, list=30%, signal=65%
REACTOME_RESOLUTION_OF_SISTER_CHROMATID_COHESION	REACTOME_RESOLUTION_OF_SISTER_CHROMATID_COHESION	30	0.689187535	3.422479517	1E-10	2.17273E-09	1.0622E-09	306	tags=90%, list=26%, signal=68%
REACTOME_MITOTIC_PROMETAPHASE	REACTOME_MITOTIC_PROMETAPHASE	35	0.661705531	3.420652125	1E-10	2.17273E-09	1.0622E-09	306	tags=86%, list=26%, signal=65%
REACTOME_SEPARATION_OF_SISTER_CHROMATIDS	REACTOME_SEPARATION_OF_SISTER_CHROMATIDS	31	0.671635839	3.380163406	1E-10	2.17273E-09	1.0622E-09	306	tags=87%, list=26%, signal=66%
REACTOME_RHO_GTPASES_ACTIVATE_FORMINS	REACTOME_RHO_GTPASES_ACTIVATE_FORMINS	27	0.679769424	3.273927618	1E-10	2.17273E-09	1.0622E-09	306	tags=89%, list=26%, signal=67%
REACTOME_SIGNALING_BY_RHO_GTPASES_MIRO_GTPASES_AND_RHOBTB3	REACTOME_SIGNALING_BY_RHO_GTPASES_MIRO_GTPASES_AND_RHOBTB3	61	0.523272352	3.238201813	1E-10	2.17273E-09	1.0622E-09	306	tags=67%, list=26%, signal=52%
REACTOME_MITOTIC_SPINDLE_CHECKPOINT	REACTOME_MITOTIC_SPINDLE_CHECKPOINT	25	0.700577862	3.292091196	1.37376E-10	2.52844E-09	1.2361E-09	306	tags=92%, list=26%, signal=69%
PID_PLK1_PATHWAY	PID_PLK1_PATHWAY	18	0.766684871	3.217426924	1.3753E-10	2.52844E-09	1.2361E-09	246	tags=94%, list=21%, signal=76%
NABA_MATRISOME	NABA_MATRISOME	126	0.392114359	2.874152689	1.84581E-09	3.15107E-08	1.54049E-08	412	tags=61%, list=36%, signal=44%
PID_AURORA_B_PATHWAY	PID_AURORA_B_PATHWAY	14	0.802198616	3.0545955	2.35613E-09	3.7541E-08	1.8353E-08	138	tags=86%, list=12%, signal=76%
REACTOME_NEURONAL_SYSTEM	REACTOME_NEURONAL_SYSTEM	41	-0.491524182	-3.496003247	8.03643E-09	1.20044E-07	5.86871E-08	288	tags=66%, list=25%, signal=51%
NABA_MATRISOME_ASSOCIATED	NABA_MATRISOME_ASSOCIATED	94	0.392571118	2.717289909	8.02299E-08	1.12794E-06	5.51426E-07	387	tags=59%, list=33%, signal=42%
REACTOME_TRANSMISSION_ACROSS_CHEMICAL_SYNAPSES	REACTOME_TRANSMISSION_ACROSS_CHEMICAL_SYNAPSES	30	-0.496856352	-3.079226809	6.62747E-07	8.79981E-06	4.30205E-06	288	tags=67%, list=25%, signal=51%
WP_OVERVIEW_OF_PROINFLAMMATORY_AND_PROFIBROTIC_MEDIATORS	WP_OVERVIEW_OF_PROINFLAMMATORY_AND_PROFIBROTIC_MEDIATORS	22	0.612274182	2.746592838	1.49319E-06	1.78436E-05	8.72336E-06	399	tags=91%, list=34%, signal=61%
REACTOME_MITOTIC_G2_G2_M_PHASES	REACTOME_MITOTIC_G2_G2_M_PHASES	22	0.612215145	2.746328004	1.49319E-06	1.78436E-05	8.72336E-06	160	tags=64%, list=14%, signal=56%
WP_CELL_CYCLE	WP_CELL_CYCLE	28	0.53878014	2.628311784	5.32655E-06	6.06212E-05	2.96364E-05	238	tags=61%, list=21%, signal=49%
WP_GASTRIC_CANCER_NETWORK_1	WP_GASTRIC_CANCER_NETWORK_1	11	0.743248892	2.563411386	5.64344E-06	6.13083E-05	2.99723E-05	246	tags=91%, list=21%, signal=72%
KEGG_CELL_CYCLE	KEGG_CELL_CYCLE	30	0.528019345	2.622124315	8.07146E-06	8.3873E-05	4.10038E-05	238	tags=60%, list=21%, signal=49%
REACTOME_EXTRACELLULAR_MATRIX_ORGANIZATION	REACTOME_EXTRACELLULAR_MATRIX_ORGANIZATION	36	0.476837599	2.510962525	8.90704E-06	8.86992E-05	4.33632E-05	387	tags=72%, list=33%, signal=50%
NABA_SECRETED_FACTORS	NABA_SECRETED_FACTORS	43	0.443766193	2.496368477	1.38549E-05	0.000132453	6.47534E-05	412	tags=70%, list=36%, signal=47%
WP_NETWORK_MAP_OF_SARSCOV2_SIGNALING_PATHWAY	WP_NETWORK_MAP_OF_SARSCOV2_SIGNALING_PATHWAY	30	0.513665193	2.550842134	2.04025E-05	0.000187546	9.16872E-05	320	tags=67%, list=28%, signal=50%
REACTOME_MITOTIC_G1_PHASE_AND_G1_S_TRANSITION	REACTOME_MITOTIC_G1_PHASE_AND_G1_S_TRANSITION	25	0.538513675	2.530534042	2.19051E-05	0.000189559	9.26715E-05	489	tags=92%, list=42%, signal=54%
REACTOME_APC_C_MEDIATED_DEGRADATION_OF_CELL_CYCLE_PROTEINS	REACTOME_APC_C_MEDIATED_DEGRADATION_OF_CELL_CYCLE_PROTEINS	14	0.662156329	2.52134534	2.22078E-05	0.000189559	9.26715E-05	238	tags=79%, list=21%, signal=63%
REACTOME_NEUROTRANSMITTER_RECEPTORS_AND_POSTSYNAPTIC_SIGNAL_TRANSMISSION	REACTOME_NEUROTRANSMITTER_RECEPTORS_AND_POSTSYNAPTIC_SIGNAL_TRANSMISSION	22	-0.513858557	-2.712535686	2.65959E-05	0.000219187	0.000107156	278	tags=68%, list=24%, signal=53%
REACTOME_VESICLE_MEDIATED_TRANSPORT	REACTOME_VESICLE_MEDIATED_TRANSPORT	42	0.438354094	2.43763836	3.62817E-05	0.000289044	0.000141308	194	tags=48%, list=17%, signal=41%
REACTOME_G2_M_CHECKPOINTS	REACTOME_G2_M_CHECKPOINTS	20	0.55408655	2.387591128	5.50571E-05	0.000424472	0.000207516	256	tags=65%, list=22%, signal=52%
REACTOME_MEMBRANE_TRAFFICKING	REACTOME_MEMBRANE_TRAFFICKING	37	0.450507885	2.4149287	6.66262E-05	0.000497614	0.000243273	194	tags=49%, list=17%, signal=42%
PID_FOXM1_PATHWAY	PID_FOXM1_PATHWAY	19	0.548973682	2.344094769	9.30396E-05	0.000673832	0.000329423	238	tags=63%, list=21%, signal=51%
KEGG_ECM_RECEPTOR_INTERACTION	KEGG_ECM_RECEPTOR_INTERACTION	15	0.601459711	2.344658095	0.000107817	0.000757889	0.000370516	303	tags=80%, list=26%, signal=60%

REACTOME_TP53_REGULATES_TRANSCRIPTION_OF_CELL_CYCLE_GENES	REACTOME_TP53_REGULATES_TRANSCRIPTION_OF_CELL_CYCLE_GENES	11	0.680154047	2.345801855	0.00011619	0.000793412	0.000387883	238	tags=82%, list=21%, signal=66%
PID_E2F_PATHWAY	PID_E2F_PATHWAY	17	0.585877717	2.41518975	0.00012839	0.000850623	0.000415852	447	tags=94%, list=39%, signal=59%
WP_RETINOBLASTOMA_GENE_IN_CANCER	WP_RETINOBLASTOMA_GENE_IN_CANCER	24	0.506920925	2.345980692	0.000131686	0.000850623	0.000415852	522	tags=92%, list=45%, signal=51%
REACTOME_TRANSCRIPTIONAL_REGULATION_BY_TP53	REACTOME_TRANSCRIPTIONAL_REGULATION_BY_TP53	31	0.462078543	2.325517627	0.000146519	0.000921525	0.000450514	441	tags=74%, list=38%, signal=47%
PID_INTEGRIN1_PATHWAY	PID_INTEGRIN1_PATHWAY	15	0.586810635	2.287551901	0.000203465	0.001246877	0.000609572	375	tags=87%, list=32%, signal=59%
REACTOME_S_PHASE	REACTOME_S_PHASE	23	0.506828098	2.310445425	0.000228848	0.001367366	0.000668477	548	tags=96%, list=47%, signal=51%
REACTOME_DNA_REPAIR	REACTOME_DNA_REPAIR	24	0.486572799	2.251811549	0.000261504	0.001524376	0.000745235	388	tags=75%, list=33%, signal=51%
REACTOME_CYCLIN_A_B1_B2_ASSOCIATED_EVENTS_DURING_G2_M_TRANSITION	REACTOME_CYCLIN_A_B1_B2_ASSOCIATED_EVENTS_DURING_G2_M_TRANSITION	10	0.672058876	2.237577065	0.000358254	0.001992611	0.000974146	238	tags=80%, list=21%, signal=64%
REACTOME_HOMOLOGY_DIRECTED_REPAIR	REACTOME_HOMOLOGY_DIRECTED_REPAIR	13	0.59383115	2.21422015	0.000358503	0.001992611	0.000974146	352	tags=85%, list=30%, signal=60%
REACTOME_ASSEMBLY_OF_COLLAGEN_FIBRILS_AND_OTHER_MULTIMERIC_STRUCTURES	REACTOME_ASSEMBLY_OF_COLLAGEN_FIBRILS_AND_OTHER_MULTIMERIC_STRUCTURES	17	0.56052696	2.310685198	0.000370462	0.002012283	0.000983763	310	tags=76%, list=27%, signal=57%
PID_SYNDECAN_1_PATHWAY	PID_SYNDECAN_1_PATHWAY	16	0.547340745	2.205116566	0.000460659	0.002446614	0.001196098	368	tags=81%, list=32%, signal=56%
REACTOME_ANTIMICROBIAL_PEPTIDES	REACTOME_ANTIMICROBIAL_PEPTIDES	10	0.659969265	2.197325481	0.000556885	0.002663894	0.001302322	55	tags=50%, list=5%, signal=48%
REACTOME_COPI_DEPENDENT_GOLGI_TO_ER_RETROGRADE_TRAFFIC	REACTOME_COPI_DEPENDENT_GOLGI_TO_ER_RETROGRADE_TRAFFIC	16	0.543423413	2.189334488	0.0005573	0.002663894	0.001302322	282	tags=69%, list=24%, signal=53%
REACTOME_GOLGI_TO_ER_RETROGRADE_TRANSPORT	REACTOME_GOLGI_TO_ER_RETROGRADE_TRANSPORT	16	0.543423413	2.189334488	0.0005573	0.002663894	0.001302322	282	tags=69%, list=24%, signal=53%
REACTOME_INTRA_GOLGI_AND_RETROGRADE_GOLGI_TO_ER_TRAFFIC	REACTOME_INTRA_GOLGI_AND_RETROGRADE_GOLGI_TO_ER_TRAFFIC	16	0.543423413	2.189334488	0.0005573	0.002663894	0.001302322	282	tags=69%, list=24%, signal=53%
REACTOME_KINESINS	REACTOME_KINESINS	16	0.543423413	2.189334488	0.0005573	0.002663894	0.001302322	282	tags=69%, list=24%, signal=53%
REACTOME_NON_INTEGRIN_MEMBRANE_ECM_INTERACTIONS	REACTOME_NON_INTEGRIN_MEMBRANE_ECM_INTERACTIONS	12	0.619873237	2.231149375	0.000584718	0.002740149	0.001339602	368	tags=92%, list=32%, signal=63%
REACTOME_DEGRADATION_OF_THE_EXTRACELLULAR_MATRIX	REACTOME_DEGRADATION_OF_THE_EXTRACELLULAR_MATRIX	22	0.490746621	2.201433923	0.000748023	0.003438029	0.001680781	368	tags=73%, list=32%, signal=51%
PID_P73PATHWAY	PID_P73PATHWAY	17	0.538185661	2.218586668	0.00078343	0.003492802	0.001707558	275	tags=65%, list=24%, signal=50%
PID_DELTA_NP63_PATHWAY	PID_DELTA_NP63_PATHWAY	13	0.572977346	2.136462502	0.000789169	0.003492802	0.001707558	398	tags=85%, list=34%, signal=56%
PID_AVB3_INTEGRIN_PATHWAY	PID_AVB3_INTEGRIN_PATHWAY	13	0.570436312	2.126987741	0.000871515	0.003778448	0.001847204	368	tags=85%, list=32%, signal=58%
REACTOME_COLLAGEN_FORMATION	REACTOME_COLLAGEN_FORMATION	20	0.490434989	2.113312852	0.000885327	0.003778448	0.001847204	387	tags=75%, list=33%, signal=51%
REACTOME_FORMATION_OF_THE_CORNIFIED_ENVELOPE	REACTOME_FORMATION_OF_THE_CORNIFIED_ENVELOPE	14	0.566760111	2.158097569	0.000955561	0.003937571	0.001924996	318	tags=71%, list=27%, signal=52%
REACTOME_KERATINIZATION	REACTOME_KERATINIZATION	14	0.566760111	2.158097569	0.000955561	0.003937571	0.001924996	318	tags=71%, list=27%, signal=52%
NABA_ECM_REGULATORS	NABA_ECM_REGULATORS	33	0.40139115	2.064650472	0.001009934	0.004091088	0.002000047	387	tags=61%, list=33%, signal=42%
REACTOME_CYTOKINE_SIGNALING_IN_IMMUNE_SYSTEM	REACTOME_CYTOKINE_SIGNALING_IN_IMMUNE_SYSTEM	63	0.325844087	2.046249642	0.001201265	0.004785037	0.002339305	619	tags=83%, list=53%, signal=41%
REACTOME_DNA_DOUBLE_STRAND_BREAK_REPAIR	REACTOME_DNA_DOUBLE_STRAND_BREAK_REPAIR	16	0.518132956	2.08744475	0.001392417	0.005455534	0.002667097	352	tags=75%, list=30%, signal=53%
REACTOME_CHROMOSOME_MAINTENANCE	REACTOME_CHROMOSOME_MAINTENANCE	12	0.592216013	2.131600958	0.001505633	0.005803972	0.002837441	352	tags=83%, list=30%, signal=59%
REACTOME_CARDIAC_CONDUCTION	REACTOME_CARDIAC_CONDUCTION	16	-0.474996842	-2.231852439	0.001543765	0.005832462	0.002851369	484	tags=94%, list=42%, signal=55%
WP_PROSTAGLANDIN_SIGNALING	WP_PROSTAGLANDIN_SIGNALING	10	0.626422625	2.085634085	0.001561831	0.005832462	0.002851369	366	tags=90%, list=32%, signal=62%
PID_MYC_ACTIV_PATHWAY	PID_MYC_ACTIV_PATHWAY	12	0.586284016	2.110249545	0.001781889	0.006389092	0.003123493	351	tags=83%, list=30%, signal=59%
REACTOME_G1_S_SPECIFIC_TRANSCRIPTION	REACTOME_G1_S_SPECIFIC_TRANSCRIPTION	10	0.62	2.064250367	0.001791084	0.006389092	0.003123493	447	tags=100%, list=39%, signal=62%
REACTOME_NEUTROPHIL_DEGRANULATION	REACTOME_NEUTROPHIL_DEGRANULATION	44	0.357913899	2.029512909	0.001779843	0.006389092	0.003123493	636	tags=86%, list=55%, signal=41%
WP_SARSCOV2_INNATE_IMMUNITY_EVASION_AND_CELLSPECIFIC_IMMUNE_RESPONSE	WP_SARSCOV2_INNATE_IMMUNITY_EVASION_AND_CELLSPECIFIC_IMMUNE_RESPONSE	13	0.539231308	2.010633541	0.002055064	0.007222947	0.003531148	366	tags=77%, list=32%, signal=53%

REACTOME_FACTORS_INVOLVED_IN_MEGAKARYOCYTE_DEVELOPMENT_AND_PLATELET_PRODUCTION	REACTOME_FACTORS_INVOLVED_IN_MEGAKARYOCYTE_DEVELOPMENT_AND_PLATELET_PRODUCTION	17	0.514399027	2.12052997	0.00212114	0.007347139	0.003591863	282	tags=65%, list=24%, signal=50%
REACTOME_SYNTHESIS_OF_DNA	REACTOME_SYNTHESIS_OF_DNA	16	0.500874126	2.017912684	0.002225244	0.00759762	0.003714317	587	tags=100%, list=51%, signal=50%
WP_BRAINDERIVED_NEUROTROPHIC_FACTOR_BDNF_SIGNALING_PATHWAY	WP_BRAINDERIVED_NEUROTROPHIC_FACTOR_BDNF_SIGNALING_PATHWAY	10	-0.571757217	-2.147623722	0.002438843	0.008209628	0.004013516	416	tags=90%, list=36%, signal=58%
REACTOME_CHEMOKINE_RECEPTORS_BIND_CHEMOKINES	REACTOME_CHEMOKINE_RECEPTORS_BIND_CHEMOKINES	10	0.606572064	2.019542911	0.002564819	0.008397146	0.004105189	366	tags=90%, list=32%, signal=62%
REACTOME_CELLULAR_SENESCENCE	REACTOME_CELLULAR_SENESCENCE	16	0.493884797	1.989754202	0.002563582	0.008397146	0.004105189	431	tags=81%, list=37%, signal=52%
WP_MALIGNANT_PLEURAL_MESOTHELIOMA	WP_MALIGNANT_PLEURAL_MESOTHELIOMA	45	0.352759462	2.017732059	0.002665252	0.008608042	0.004208292	395	tags=60%, list=34%, signal=41%
WP_DNA_IRDAMAGE_AND_CELLULAR_RESPONSE_VIA_ATR	WP_DNA_IRDAMAGE_AND_CELLULAR_RESPONSE_VIA_ATR	17	0.504723654	2.08064475	0.002798585	0.008918158	0.004359901	497	tags=88%, list=43%, signal=51%
REACTOME_DNA_REPLICATION	REACTOME_DNA_REPLICATION	18	0.46381685	1.946427894	0.00285897	0.008990708	0.004395369	587	tags=94%, list=51%, signal=47%
KEGG_OOCYTE_MEIOSIS	KEGG_OOCYTE_MEIOSIS	20	0.453529714	1.954285882	0.003046547	0.009456164	0.004622921	238	tags=60%, list=21%, signal=49%
REACTOME_ACTIVATION_OF_NMDA_RECEPTORS_AND_POSTSYNAPTIC_EVENTS	REACTOME_ACTIVATION_OF_NMDA_RECEPTORS_AND_POSTSYNAPTIC_EVENTS	13	-0.502419916	-2.18764041	0.003172263	0.00972014	0.004751973	278	tags=69%, list=24%, signal=53%
REACTOME_INTEGRIN_CELL_SURFACE_INTERACTIONS	REACTOME_INTEGRIN_CELL_SURFACE_INTERACTIONS	12	0.562581481	2.024935494	0.003414804	0.010330862	0.005050542	368	tags=83%, list=32%, signal=57%
REACTOME_MHC_CLASS_II_ANTIGEN_PRESENTATION	REACTOME_MHC_CLASS_II_ANTIGEN_PRESENTATION	16	0.481705113	1.940684908	0.00426323	0.01257916	0.006149688	282	tags=62%, list=24%, signal=48%
KEGG_CYTOKINE_CYTOKINE_RECEPTOR_INTERACTION	KEGG_CYTOKINE_CYTOKINE_RECEPTOR_INTERACTION	36	0.356494954	1.877254378	0.004240116	0.01257916	0.006149688	547	tags=78%, list=47%, signal=42%
NABA_COLLAGENS	NABA_COLLAGENS	13	0.515019765	1.920355882	0.004629395	0.013330428	0.006516968	368	tags=77%, list=32%, signal=53%
REACTOME_COLLAGEN_CHAIN_TRIMERIZATION	REACTOME_COLLAGEN_CHAIN_TRIMERIZATION	13	0.515019765	1.920355882	0.004629395	0.013330428	0.006516968	368	tags=77%, list=32%, signal=53%
REACTOME_RHOA_GTPASE_CYCLE	REACTOME_RHOA_GTPASE_CYCLE	12	0.543503846	1.956268143	0.005358153	0.015065865	0.007365387	377	tags=83%, list=32%, signal=57%
WP_BURN_WOUND_HEALING	WP_BURN_WOUND_HEALING	18	0.447547991	1.878154909	0.00532445	0.015065865	0.007365387	508	tags=83%, list=44%, signal=48%
REACTOME_ECM_PROTEOGLYCANS	REACTOME_ECM_PROTEOGLYCANS	13	0.511119105	1.905811479	0.005516456	0.015330615	0.007494817	368	tags=77%, list=32%, signal=53%
REACTOME_SIGNALING_BY_INTERLEUKINS	REACTOME_SIGNALING_BY_INTERLEUKINS	41	0.359307906	1.975494418	0.005729591	0.01573991	0.007694913	611	tags=85%, list=53%, signal=42%
REACTOME_INTEGRATION_OF_ENERGY_METABOLISM	REACTOME_INTEGRATION_OF_ENERGY_METABOLISM	11	-0.490386126	-1.966745119	0.006796946	0.018459886	0.009024653	288	tags=73%, list=25%, signal=55%
REACTOME_REGULATION_OF_TP53_ACTIVITY	REACTOME_REGULATION_OF_TP53_ACTIVITY	15	0.485056785	1.890886947	0.007432781	0.019828483	0.009693731	139	tags=47%, list=12%, signal=42%
REACTOME_HDR_THROUGH_HOMOLOGOUS_RECOMBINATION_HRR	REACTOME_HDR_THROUGH_HOMOLOGOUS_RECOMBINATION_HRR	10	0.559373891	1.862399613	0.007466793	0.019828483	0.009693731	434	tags=90%, list=37%, signal=57%
REACTOME_TRANSPORT_OF_SMALL_MOLECULES	REACTOME_TRANSPORT_OF_SMALL_MOLECULES	69	-0.208563926	-1.763065528	0.00767777	0.020164692	0.009858096	279	tags=42%, list=24%, signal=34%
NABA_CORE_MATRISOME	NABA_CORE_MATRISOME	32	0.358035288	1.830150715	0.007947094	0.020645167	0.010092991	406	tags=62%, list=35%, signal=42%
PID_ATR_PATHWAY	PID_ATR_PATHWAY	11	0.53907275	1.85922272	0.008226566	0.020984102	0.010258689	158	tags=55%, list=14%, signal=48%
WP_P53_TRANSCRIPTIONAL_GENE_NETWORK	WP_P53_TRANSCRIPTIONAL_GENE_NETWORK	16	0.457766347	1.844240841	0.008253161	0.020984102	0.010258689	318	tags=62%, list=27%, signal=46%
REACTOME_PROTEIN_PROTEIN_INTERACTIONS_AT_SYNAPSES	REACTOME_PROTEIN_PROTEIN_INTERACTIONS_AT_SYNAPSES	11	-0.485332022	-1.946475106	0.00850953	0.021408186	0.010466015	409	tags=82%, list=35%, signal=53%
PID_FRA_PATHWAY	PID_FRA_PATHWAY	12	0.515702635	1.856201465	0.009095268	0.022643428	0.011069899	368	tags=75%, list=32%, signal=52%
REACTOME_COLLAGEN_DEGRADATION	REACTOME_COLLAGEN_DEGRADATION	17	0.46759966	1.92760686	0.009199372	0.022666493	0.011081175	368	tags=71%, list=32%, signal=49%
REACTOME_COLLAGEN_BIOSYNTHESIS_AND_MODIFYING_ENZYMES	REACTOME_COLLAGEN_BIOSYNTHESIS_AND_MODIFYING_ENZYMES	15	0.472090222	1.840339661	0.009711408	0.023683943	0.011578585	387	tags=73%, list=33%, signal=50%
REACTOME_SWITCHING_OF_ORIGINS_TO_A_POST_REPLICATIVE_STATE	REACTOME_SWITCHING_OF_ORIGINS_TO_A_POST_REPLICATIVE_STATE	10	0.546086957	1.818161613	0.010592373	0.025433598	0.012433954	532	tags=100%, list=46%, signal=55%
WP_G1_TO_S_CELL_CYCLE_CONTROL	WP_G1_TO_S_CELL_CYCLE_CONTROL	13	0.480415416	1.791326531	0.010641673	0.025433598	0.012433954	476	tags=85%, list=41%, signal=50%

REACTOME_ACTIVATION_OF_ATR_IN_RESPONSE_TO_REPLICATION_STRESS	REACTOME_ACTIVATION_OF_ATR_IN_RESPONSE_TO_REPLICATION_STRESS	10	0.542443169	1.806029856	0.011286949	0.02644687	0.012929322	124	tags=50%, list=11%, signal=45%
REACTOME_INTERLEUKIN_10_SIGNALING	REACTOME_INTERLEUKIN_10_SIGNALING	11	0.523338923	1.804957893	0.011176743	0.02644687	0.012929322	428	tags=82%, list=37%, signal=52%
REACTOME_METABOLISM_OF_CARBOHYDRATES	REACTOME_METABOLISM_OF_CARBOHYDRATES	17	0.458020444	1.888118035	0.011939089	0.027703323	0.013543576	315	tags=65%, list=27%, signal=48%
KEGG_P53_SIGNALING_PATHWAY	KEGG_P53_SIGNALING_PATHWAY	14	0.488527904	1.860206572	0.012103893	0.027815677	0.013598503	441	tags=79%, list=38%, signal=49%
PID_TAP63_PATHWAY	PID_TAP63_PATHWAY	12	0.506266125	1.82223603	0.012672976	0.028846107	0.014102259	119	tags=42%, list=10%, signal=38%
REACTOME_INNATE_IMMUNE_SYSTEM	REACTOME_INNATE_IMMUNE_SYSTEM	77	0.257303148	1.685831065	0.013137586	0.029621537	0.01448135	636	tags=75%, list=55%, signal=36%
REACTOME_HEMOSTASIS	REACTOME_HEMOSTASIS	49	0.305946845	1.78661431	0.013390807	0.029910308	0.014622524	282	tags=43%, list=24%, signal=34%
KEGG_FOCAL_ADHESION	KEGG_FOCAL_ADHESION	16	0.432996214	1.744447371	0.013629538	0.030161663	0.014745407	303	tags=62%, list=26%, signal=47%
WP_DNA_DAMAGE_RESPONSE	WP_DNA_DAMAGE_RESPONSE	13	0.470995913	1.756204	0.013997759	0.030413313	0.014868433	238	tags=54%, list=21%, signal=43%
WP_MIRNA_REGULATION_OF_DNA_DAMAGE_RESPONSE	WP_MIRNA_REGULATION_OF_DNA_DAMAGE_RESPONSE	13	0.470995913	1.756204	0.013997759	0.030413313	0.014868433	238	tags=54%, list=21%, signal=43%
WP_DNA_REPAIR_PATHWAYS_FULL_NETWORK	WP_DNA_REPAIR_PATHWAYS_FULL_NETWORK	12	0.492883127	1.774065751	0.018280363	0.03900899	0.01907068	388	tags=75%, list=33%, signal=50%
WP_IL18_SIGNALING_PATHWAY	WP_IL18_SIGNALING_PATHWAY	22	0.385748847	1.730425764	0.018257512	0.03900899	0.01907068	428	tags=68%, list=37%, signal=44%
REACTOME_ADAPTIVE_IMMUNE_SYSTEM	REACTOME_ADAPTIVE_IMMUNE_SYSTEM	48	0.291851401	1.696994567	0.018840095	0.039847634	0.019480676	282	tags=40%, list=24%, signal=31%
REACTOME_CLASS_I_MHC_MEDIATED_ANTIGEN_PROCESSING_PRESENTATION	REACTOME_CLASS_I_MHC_MEDIATED_ANTIGEN_PROCESSING_PRESENTATION	12	0.486202033	1.750018063	0.022317698	0.046788859	0.022874095	31	tags=33%, list=3%, signal=33%
